# Biogeography of the ecosystems of the healthy human body

**DOI:** 10.1186/gb-2013-14-1-r1

**Published:** 2013-01-14

**Authors:** Yanjiao Zhou, Hongyu Gao, Kathie A Mihindukulasuriya, Patricio S La Rosa, Kristine M Wylie, Tatiana Vishnivetskaya, Mircea Podar, Barb Warner, Phillip I Tarr, David E Nelson, J Dennis Fortenberry, Martin J Holland, Sarah E Burr, William D Shannon, Erica Sodergren, George M Weinstock

**Affiliations:** 1 The Genome Institute, Washington University School of Medicine, St. Louis, MO, 63108 USA; 2 Department of Medicine, Division of General Medical Sciences, Washington University School of Medicine, St. Louis, MO, 63110 USA; 3 University of Tennessee, Knoxville, TN, 37916 USA; 4 Oak Ridge National Laboratory, Oak Ridge, TN, 37831 USA; 5 Department of Pediatrics, Division of Newborn Medicine, Washington University School of Medicine, St. Louis, MO, 63110 USA; 6 Department of Pediatrics, Division of Pediatric Gastroenterology, Washington University School of Medicine, St. Louis, MO, 63110 USA; 7 Department of Biology, Indiana University, Bloomington, IN, 47405 USA; 8 Section of Adolescent Medicine, Department of Pediatrics, Indiana University School of Medicine, Indianapolis, IN, 46202 USA; 9 London School of Hygiene and Tropical Medicine, London, WC1E 7HT United Kingdom; 10 Medical Research Council Unit, The Gambia, Fajara, 1000 The Gambia

**Keywords:** Biogeography, Human microbiome, Biodiversity, Temporal stability

## Abstract

**Background:**

Characterizing the biogeography of the microbiome of healthy humans is essential for understanding microbial associated diseases. Previous studies mainly focused on a single body habitat from a limited set of subjects. Here, we analyzed one of the largest microbiome datasets to date and generated a biogeographical map that annotates the biodiversity, spatial relationships, and temporal stability of 22 habitats from 279 healthy humans.

**Results:**

We identified 929 genera from more than 24 million 16S rRNA gene sequences of 22 habitats, and we provide a baseline of inter-subject variation for healthy adults. The oral habitat has the most stable microbiota with the highest alpha diversity, while the skin and vaginal microbiota are less stable and show lower alpha diversity. The level of biodiversity in one habitat is independent of the biodiversity of other habitats in the same individual. The abundances of a given genus at a body site in which it dominates do not correlate with the abundances at body sites where it is not dominant. Additionally, we observed the human microbiota exhibit both cosmopolitan and endemic features. Finally, comparing datasets of different projects revealed a project-based clustering pattern, emphasizing the significance of standardization of metagenomic studies.

**Conclusions:**

The data presented here extend the definition of the human microbiome by providing a more complete and accurate picture of human microbiome biogeography, addressing questions best answered by a large dataset of subjects and body sites that are deeply sampled by sequencing.

## Background

Biogeography in traditional ecology attempts to describe spatial and temporal patterns of biological diversity of organisms [1]. It seeks to answer what organisms are present, how they are distributed, and how they vary over time. Biogeographical patterns allow one to explore what ecological rules apply, such as cosmopolitan (global) or endemic (confined to a location) distribution patterns of taxa.

The human body is composed of many niches that are directly or indirectly exposed to the external environment and are modulated by interaction with multiple systems such as the host immune, endocrine, and nervous systems. Each niche thus serves as a unique and complex space for microbes to survive and thrive. Alteration of the microbial balance in humans is associated with disease [2-12]. Characterization of the human microbial community by culture-independent, high-throughput sequencing unveiled the high diversity of microbes in different habitats of the body. The gut microbiome is the best-characterized human microbial habitat, and it was originally estimated to harbor 500 to 1,000 species [13]. Bacterial community structures in other habitats of the human body such as the oral, skin, nasal, and vaginal areas also have been addressed [14-21]. Over 500 species-level phylotypes were discovered in oral habitats [16,22]. Two hundred and five genera were identified on the skin surface from 10 subjects [18]. Traditionally, urine has been thought to be sterile but culture-independent sequencing uncovered diverse microbial communities and substantial intra- and inter-subject variability [7].

High variation of microbial composition between individuals requires deeper sequencing and larger subject numbers to have a more complete view of human microbiome. The NIH launched the Human Microbiome Project (HMP) [23], aiming to more fully characterize the human microbiota and address its role in health and disease. This project enrolled 300 healthy, young adults, collected samples from 15 (male) or 18 (female) habitats in the body, and produced datasets of both 16S rRNA gene and whole genome shotgun (WGS) sequences [24]. The 16S rRNA gene sequences aim to define community structures in different habitats, representing major niches of the human body, including stool, oral, skin, nasal, and vaginal areas. The HMP consortium has been conducting extensive analysis with the HMP 16s rRNA gene dataset to explore different aspects of human microbiome [24-29]. RDP-based taxonomic, operational taxonomic unit (OTU), and phylogenetic approaches are the main analytic methods used to characterize microbial community structure [24,25,28]. The RDP taxonomic approach used in this study possesses easy interpretability and better sequence error tolerance. It provides confident taxonomic assignment at the genus and other higher taxonomic levels. The OTU approach offers a sub-genus level resolution. However, the OTU does not always reflect a biological unit, and technical factors (single, average, complete linkage) largely affect the components within an OTU. The phylogenetic approach dependent on the tree construction provides phylogeny of the bacterial community, but it inevitably bears the intrinsic problems of tree construction using short reads. Each of these methods has its pros and cons, and they are complementary to each other. They provide equally important insight into the bacterial community structure [30].

As a companion paper of the HMP main papers, we presented an extensive taxonomic analysis of the majority of the HMP samples as well as additional samples comprising preterm baby stool, two urogenital sites, and the conjunctiva in adults, thus expanding the view of the microbiome beyond that of the HMP. We addressed the following aspects of human microbial biogeography in the context of a large cohort and deep sequencing: how many organisms inhabit the human body; is the biodiversity of one habitat influenced by other habitats; is the presence or abundance of the organism in one habitat affected by other habitats; what is the bacterial distribution pattern in a habitat; what is the degree of inter-personal variation and temporal variation in different habitats; are the HMP data comparable with the prior human microbiome studies. The analysis focusing on the above questions are not addressed in other HMP companion papers or are insufficiently investigated in the main papers. Answering these questions allows us to have a more complete and broad understanding of the ecosystems of the human body.

## Results

### Overview of the datasets

Sample collection, DNA extraction, sequencing, as well as data processing followed the manual of procedures of the HMP consortium [24]. Based on investigations of mock communities and a pilot study of 24 subjects from the HMP, we found bacterial community structure, including composition and abundance, was not biased by the center performing sequencing and there was consistency of community structure between technical replicates [24,25,31].

To have a broader view of the human microbiome, we used datasets from the HMP and healthy controls from two HMP demonstration projects, necrotizing enterocolitis (neonatal stool) and urethritis microbiome (urine and penis) as well as a study of the conjunctiva. All subjects were adults except for necrotizing enterocolitis (preterm babies) and urethral microbiome (adolescents). All samples were from the USA except conjunctiva (The Gambia). The description of each dataset is summarized in Table 1.

**Table 1:** Cumulative and average number of taxa per body habitat

Total taxa									Mean ± SE						
**Body habitat**	**Project**	**Subjects (*n*)**	**Reads**	**Phylum**	**Class**	**Order**	**Family**	**Genus**	**Reads (m ± sd)**	**Phylum**	**Class**	**Order**	**Family**	**Genus**	**Genus_1000^a^**

**Gut**															
Stool	HMP	209	1774406	13	23	38	76	203	8490 ± 8308.1	6 ± 0.1	10 ± 0.2	12 ± 0.3	25 ± 0.5	54 ± 1.2	32 ± 0.7
Stool (preterm)	NEC	10	84574	6	12	21	39	90	8457 ± 3505.8	4 ± 0.3	5 ± 0.4	7 ± 0.6	12 ± 1.1	26 ± 2.5	14 ± 0.7
**Nasal**															
Anterior nares	HMP	166	1178682	20	38	75	165	457	7100 ± 4031.8	7 ± 0.1	12 ± 0.2	17 ± 0.3	34 ± 0.8	53 ± 1.5	27 ± 0.9
**Oral**															
Buccal mucosa	HMP	186	1542387	15	25	46	99	241	8292 ± 5166.5	7 ± 0.1	12 ± 0.2	17 ± 0.2	33 ± 0.5	59 ± 1.1	33 ± 0.7
Hard palate	HMP	193	1551365	19	34	62	127	301	8038 ± 4518.7	7 ± 0.1	13 ± 0.2	18 ± 0.3	36 ± 0.5	63 ± 1	39 ± 0.6
Keratinized gingiva	HMP	199	1553381	13	22	37	88	218	7806 ± 4591.9	6 ± 0.1	11 ± 0.1	14 ± 0.2	25 ± 0.4	41 ± 0.9	23 ± 0.5
Palatine tonsil	HMP	199	1648985	15	25	47	93	242	8286 ± 5068.5	8 ± 0.1	14 ± 0.2	18 ± 0.3	35 ± 0.5	62 ± 1	40 ± 0.6
Saliva	HMP	182	1335119	17	29	49	99	266	7336 ± 4083.7	9 ± 0.1	15 ± 0.2	20 ± 0.3	39 ± 0.5	72 ± 0.9	50 ± 0.6
Subgingival plaque	HMP	201	1603380	14	23	42	84	244	7977 ± 4364.2	8 ± 0.1	14 ± 0.2	19 ± 0.2	38 ± 0.4	68 ± 0.9	46 ± 0.6
Supragingival plaque	HMP	208	1677580	15	24	40	77	219	8065 ± 4539.6	8 ± 0.1	13 ± 0.2	17 ± 0.3	35 ± 0.5	62 ± 0.9	41 ± 0.7
Throat	HMP	186	1455561	18	29	53	119	322	7826 ± 3784.6	8 ± 0.1	13 ± 0.2	18 ± 0.3	36 ± 0.5	63 ± 1	41 ± 0.6
Tongue dorsum	HMP	206	1769242	13	21	35	74	185	8589 ± 7921.7	7 ± 0.1	13 ± 0.2	16 ± 0.2	32 ± 0.4	56 ± 0.8	35 ± 0.5
**Skin**															
Antecubital fossa (left)	HMP	75	470729	21	41	78	168	501	6276 ± 4981.6	8 ± 0.2	13 ± 0.4	23 ± 0.7	48 ± 1.7	81 ± 3.9	56 ± 2.5
Antecubital fossa (right)	HMP	87	541278	21	42	81	186	534	6222 ± 5348.3	8 ± 0.2	14 ± 0.4	23 ± 0.8	48 ± 1.9	80 ± 3.9	52 ± 2.4
Retroauricular crease (left)	HMP	181	1762310	20	38	75	162	474	9737 ± 6158.1	7 ± 0.1	11 ± 0.2	17 ± 0.4	31 ± 1	47 ± 1.9	19 ± 1.0
Retroauricular crease (right)	HMP	189	1710301	23	42	79	173	484	9049 ± 4839.4	6 ± 0.1	11 ± 0.2	16 ± 0.4	30 ± 0.9	44 ± 1.7	19 ± 1.0
**Ocular**															
Conjunctiva	The Gambia	15	158577	20	37	70	165	409	10571 ± 10554.	8 ± 0.8	14 ± 0.9	21 ± 1.4	37 ± 2.8	81 ± 8.7	54 ± 5.7
**Urogenital**															
Mid vagina	HMP	89	732322	10	19	36	75	218	8228 ± 6675.7	5 ± 0.1	8 ± 0.2	11 ± 0.4	20 ± 0.8	29 ± 1.5	12 ± 0.9
Posterior fornix	HMP	89	822111	10	18	31	70	176	9237 ± 7652	5 ± 0.1	7 ± 0.2	9 ± 0.4	15 ± 0.7	20 ± 1.2	7 ± 0.6
Vaginal introitus	HMP	80	660867	13	20	35	75	203	8261 ± 4389.5	5 ± 0.1	9 ± 0.2	12 ± 0.4	23 ± 0.8	34 ± 1.5	16 ± 0.9
Urine	Urethritis	18	152360	23	38	64	131	312	8464 ± 4694.9	9 ± 0.5	14 ± 1.4	24 ± 2.5	49 ± 5.6	61 ± 5.2	38 ± 4.8
Penis	Urethritis	18	203866	20	35	64	135	333	11326 ± 3523.4	8 ± 0.7	14 ± 1	22 ± 1.9	42 ± 3.3	68 ± 5.5	30 ± 3.0
**Total**	All projects	279	24385112	30	109	125	493	929	8173 ± 5556	ND	ND	ND	ND	ND	ND

^a^1,000 reads were subsampled from each sample and the average number of genera is listed.

Over 24 million, high-quality 16S rRNA gene sequences (generated on the Roche-454 Titanium FLX platform) were included in the analysis. All sequences were generated from the V3-V5 variable regions of the 16S rRNA gene with the exception of conjunctiva (V1-V3, due to the better amplification of V1-V3 of conjunctiva samples). The reads were from 2,983 specimens, sampled from 236 HMP healthy subjects at 15 (male) or 18 (female) body habitats, 18 subjects at urine and penis habitats, 10 preterm babies at the stool habitat, and 15 subjects at the conjunctiva (Table 1). Raw reads were processed by filtering low quality reads and removing chimeras as described by the HMP [24,25,31]. The average read length of V3-V5 was 468 ± 82 bp, and the average sequencing depth was 8,167 reads ± 5,556 reads (mean ± sd). All reads were further classified by the RDP classifier [32] (version 2.2 with training set 6) from phylum to genus level at the 50% confidence threshold.

### Biodiversity of human microbiota

One of the goals of the HMP is to characterize the bacterial composition and distribution pattern in and on the human body. In the combined extended datasets in the present analysis, we identified 30 phyla, 51 classes, 125 orders, 493 families, and 929 genera from all body habitats (Table 1). In addition, a wide range of unclassified organisms was present in each habitat (Figure S1 in Additional file 1), from which novel organisms at the genus level or higher rank were identified [33].

The 30 bacterial phyla observed represent less than half of the known bacterial phyla [34]. The human habitats contain a large number of *Firmicutes, Actinobacteria, Proteobacteria*, and *Bacterioidetes *(Figure S2A in Additional file 1). *Actinobacteria *and *Proteobacteria *were the most predominant phyla in the marine and soil microbiomes [35,36] whereas *Firmicutes *were less dominant, indicating a major difference between the external environments, the presumed source of microbes, and the human body. Bacterial distributions at the phylum level exhibited different patterns in different habitats. The differences were revealed in both abundant phyla as well as low abundance phyla (<0.5% of the average relative abundance) (Figure S2A, S2B in Additional file 1).

**Figure 1:**
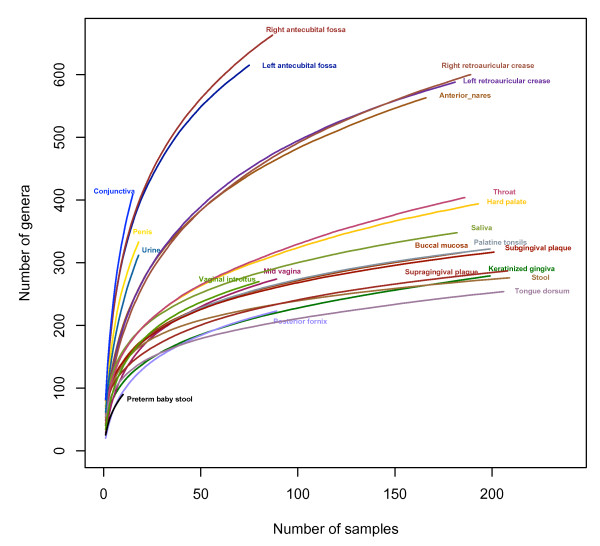
**Accumulation curves of 22 habitats**. Each line represents the accumulative richness from all subjects. All the reads were included in the analysis to have a full view of the genera revealed by the HMP and other datasets. At the genus level, oral, vaginal, and stool habitats become asymptotically flat at the current sampling depth and sampling efforts. More subjects are needed to reach the saturation for skin and skin-associated habitats.

At the genus level we identified 501 and 534 genera from left and right antecubital fossa, respectively, and 474 and 484 genera from left and right retroauricular crease, respectively, making these four skin sites the richest communities even though the number of subjects sequenced from skin habitats was less than oral and stool habitats. As indicated by the accumulation curves in Figure 1, skin and skin-associated sites continue to yield more taxa with increasing numbers of subjects. On the other hand, the slopes of the accumulation curves for the three vaginal sites, seven of nine oral sites (except throat and hard palate), and the stool become asymptotically flatter, suggesting the sampling is close to saturation for those habitats. This indicates a lower richness of genera in these sites.

The accumulation curves in Figure 1 represent the overall richness of each habitat, which is a function of both sequence depth and sample size. Accumulation curves based on subsampling down to 1,000 reads show similar patterns as using all of the reads, with skin being the richest community (Table 1, Figure S3A in Additional file 1).

**Figure 2:**
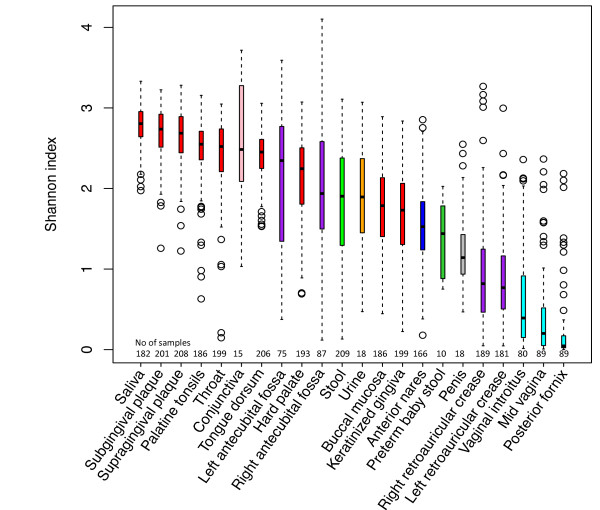
**Shannon diversity comparisons of 22 habitats**. One thousand reads were rarefied from each sample. The boxplot shows the minimum, 25th percentile, median, 75th percentile, and the maximum of the data from bottom to top. Oral habitats in general have more even bacterial communities, and vaginal habitats have the lowest Shannon diversity.

Richness is one dimension of biodiversity. Shannon diversity is another diversity index that measures both the richness and evenness. To compare the differences of diversity between habitats, a t-test was performed for each combination of two different habitats. *P *values corrected by Bonferroni method are summarized in Table S1 in Additional file 2. Oral sites, particularly saliva, have the highest evenness (Figure 2). Buccal mucosa and keratinized gingiva have lower diversity than the other seven oral sites. Posterior fornix of the vagina shows the lowest diversity. Stool and skin sites show moderate diversity.

In contrast to the overall richness estimated at the genus level, the stool habitat had the highest species (OTU) level richness (with an OTU defined as 97% identity of sequences), followed by oral, skin, and vaginal habitats [28]. Stool, the best-studied habitat, was ranked second to last in terms of community richness at the genus level among all of the 18 HMP habitats. The richness difference observed in genus and OTU level may be because: (1) the richness observed at genus level is database-dependent (RDP database); or (2) there are more unclassified genera in stool and oral sites than skin and vaginal sites (Figure S1 in Additional file 1), and the unclassified genera may contain different OTUs.

### Universal distribution pattern of human microbiota

**Figure 3:**
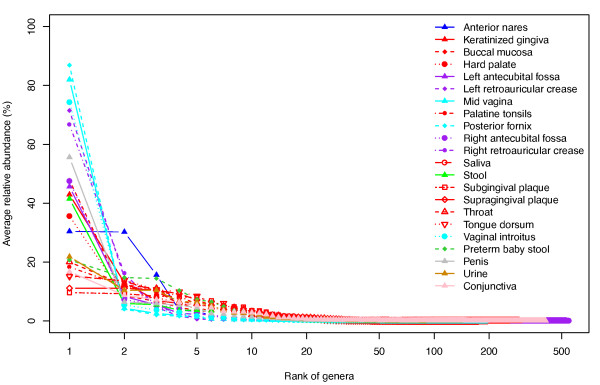
**Bacterial distribution patterns viewed by rank abundance curves**. The genus distributions are illustrated by rank abundance curves. The x-axis represents the ranked genera from high to low. The y-axis shows the average relative abundance for a given genus. Twenty-two different line shapes and nine different colors represent the 22 habitats in this study. One or a few genera dominate each habitat with a long tail representing rare genera. This bacterial distribution pattern agrees with the species abundance pattern in other environments.

The bacterial distribution patterns in all 22 human habitats follow a general rule, which is seen in other systems: all communities are dominated by from one to several genera, and rank abundance curves (RAC) have a long tail of less abundant organisms (Figure 3). These minor organisms are detected after quality filtering and chimera removal and thus are unlikely to be due to these types of artifacts. Concern has been previously raised over the possibility that minor species/OTUs are nevertheless 'noise' due to various issues. One of these is sequencing artifacts that are not removed by quality-based filtering [37,38]. As noted earlier, our methods were developed using mock community approaches, in which a limited number of false taxa were detected [31]. Moreover, different from the studies at the species/OTU level, analysis at the genus level is also less likely to be as sensitive to false taxon calls due to small sequence errors. Thus we do not consider sequencing errors to be a major source of the minor organisms detected.

**Figure 4:**
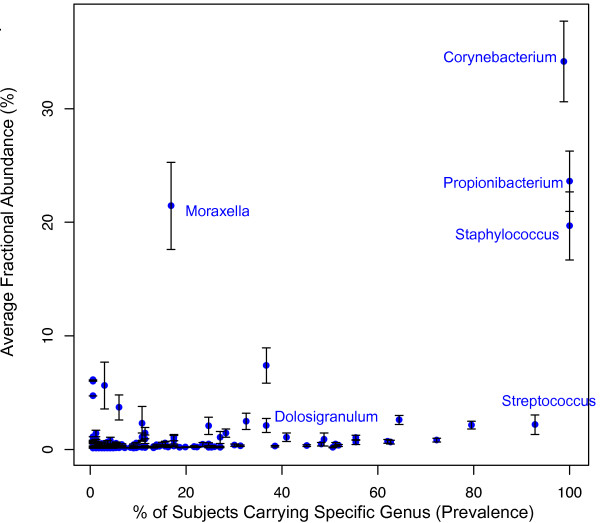
**Cosmopolitan and endemic aspects of human microbiota**. The relative abundance and prevalence of each taxon from anterior nares is plotted to indicate the cosmopolitan and endemic features of human microbiota. Each dot represents a genus. The x-axis represents the fraction of subjects carrying a given genus (prevalence). The y-axis shows the average fractional abundance (m ± se) of that genus in those subjects. In general, highly abundant genera tend to be found in more subjects while lower abundance genera are less widely distributed. However, some high abundance genera are harbored by only a subset of subjects

As described previously, the best evidence for the genuine existence of a minor taxon is its appearance across many distinct samples [38]. If samples are truly distinct, the possibility of contamination as the source of minor taxa that are prevalent among samples is reduced. The HMP samples were collected separately in St. Louis and Houston and sequenced at four sequencing centers, and may thus be regarded as such distinct data sources. We note below that genera that are present at low abundances, for instance nasal *Streptococcus *have an average relative abundance of 2%, but are found in >80% of the nasal samples. Two-thirds of those samples with *Streptococcus *are from Houston, and sequenced roughly evenly at the four sequencing centers, making it unlikely to be noise from contaminants (Figure 4).

There are other organisms that are present at low abundance but are found in few samples. To further explore the prevalence of these minor organisms, we chose 60 stool samples that had >9,000 reads and subsampled 1,000, 3,000, 6,000, and 9,000 reads from these samples. At each sequencing depth, the prevalence of each taxon was calculated (Figure S4 in Additional file 1). With increasing sequencing depth, the prevalence among the subjects for the majority of the taxa at the 1,000 read depth increased 3.6-fold on average. As an example of a well-known minor genus, that is not a false taxon, we analyzed *Escherichia/Shigella*, and found it in five out of 60 subjects at 1,000 reads, and increased to 24 subjects at 9,000 reads. As a second example, we chose *Corynebacterium *and *Propionibacterium*, which make up the majority of skin microbiota, but are not a major part of the fecal flora. They were each identified in one subject at 1,000 read depth and increased to 11 and six subjects, respectively, at 9,000 reads. These examples show that there are likely real taxa among the low abundance genera that are not as prevalent among subjects.

### Diversity correlation between major habitats from the same subjects

**Figure 5:**
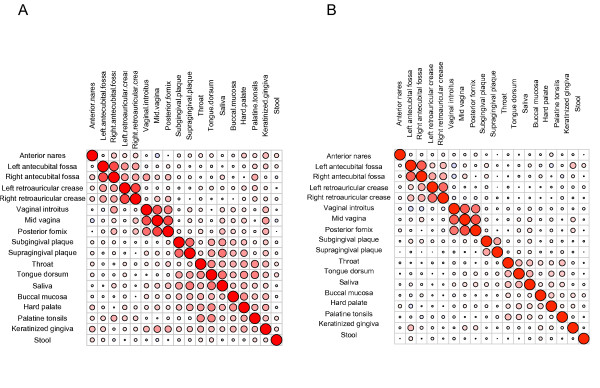
**Biodiversity correlation between habitats**. The symmetric plot was used to show the association of biodiversity between paired habitats. The size and the redness of the circle represent the degree of correlation. Large size and deep redness indicate strong correlation. The large red circles on the diagonal line represent self-comparisons. Proximal habitats have similar alpha and beta diversity. (**A**) Alpha diversity association. The association of bacterial richness of different habitats from the same individuals is expressed by the Spearman correlation coefficient. (**B**) Beta diversity association. Mantel correlation was used to compare the Bray-Curtis dissimilarity matrix.

Many previous studies addressed the bacterial community biodiversity of a single habitat, thus biodiversity correlations between habitats were not described. To answer this question, we chose two different body sites from the same subjects and computed their richness. Correlation of body sites' richness within a subject was then expressed by the Spearman correlation coefficient. The richness correlation was 0.68 between subgingival plaque and supragingival plaque, 0.66 between left and right retroauricular crease, 0.68 between left and right antecubital fossa, and 0.41 to 0.68 within vaginal sites, indicating a stronger correlation for those pairs. In contrast, correlations between skin and oral, skin and vaginal, or skin and stool sites were very low, indicating richness was not correlated (Figure 5A). Therefore, similar body sites had stronger correlation of richness, while dissimilar sites had little or no correlation. More specifically, an individual who harbors a greater collection of taxa in saliva is more likely to have more taxa on the tongue, but not necessarily more taxa in skin, stool, or vagina. It has been well known that major habitats are distinctive by their specific dominant taxa [25,39], and a finer distinction between similar habitats, such as oral sites, is achievable at the OTU level [28]. The taxa preference to certain habitats is generally explained by environmental selection. The richness specificity for the major habitats that we found here contributed to another interesting ecological observation.

Beta diversity, as measured by the Bray-Curtis dissimilarity index, also correlated well for similar body sites and weakly for dissimilar body sites (Figure 5B). That is, two individuals who have similar bacterial communities in left and right retroauricular creases show similar communities in their antecubital fossae. This result does not provide support for the possibility that host genotype is the major modulator of microbial content since no individuals were observed with higher diversity in all body sites, as might be expected if a host mechanism controlling microbiome diversity were a major factor. However, the sample size and other factors such as environmental determinants may obscure smaller effects of the host.

### Quantitative measurement of bacterial community variation

Understanding the variation of bacterial communities between healthy subjects is a prerequisite to investigating the role of microbiota in health and disease. Previous studies of fewer subjects revealed high interpersonal variation of bacterial community structure. Because this study features more subjects and deep sequencing than previous studies, we are able to more accurately assess this variation. Here, we evaluated the interpersonal variation at the single taxon level as well as the whole community level.

The variation of a single taxon in a habitat was measured by the range and quantiles of the taxon relative abundance. The quantiles of the top 20 most abundant genera in each site are summarized in Table S2 in Additional file 2, which provides a reference on bacterial variation of healthy subjects for future studies. The taxon variation in vaginal sites was evident by the wide range of *Lactobacillus *(0-100%). In particular, at 4,000 read depth, the *Lactobacillus *genus was not detected in some subjects, and was the only genus detected in other subjects. This large variation of dominant genera was also seen in four skin sites, anterior nares, and penis samples, and in stool samples where the relative abundance of *Bacteroides *ranged from 1.3% to 98.2%.

Oral sites showed a more even abundance of major organisms compared to skin and vaginal sites (Figure 2), and associated with this, the genera in oral habitats have a narrower range of abundances. For instance, the relative abundance of *Prevotella *in saliva ranges from 2.3% to 47.4%.

**Figure 6:**
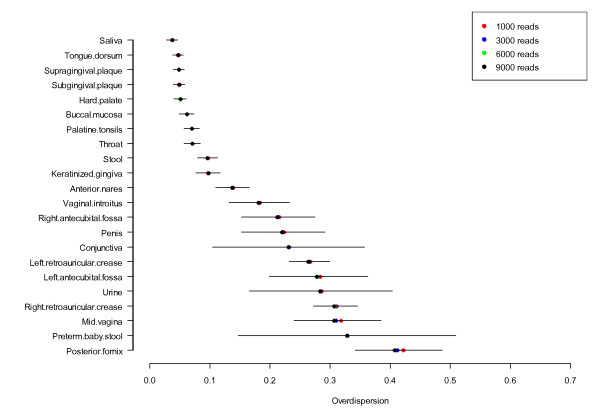
**Bacterial community variation**. Overall the variation of bacterial community for each habitat was evaluated by the over-dispersion parameter theta (m ± sd) from the Dirichlet-multinomial model. Higher theta indicates higher variation and *vice versa*. The variation within a bacterial community was calculated at 1,000, 3,000, 6,000, and 9,000 read depths as shown by different colors. No significant difference was found in bacterial community variation at different read depths.

While variation of the abundance of a single taxon between subjects is important, the variation of the abundance of the whole bacterial composition is another important measure. The HMP consortium not only produced a large amount of sequence data, but it also developed new analytical tools to cope with the high dimensional metagenomic data [40,41]. Given the multivariate nature of the metagenomic data, we recently developed multivariate statistical method, Dirichlet-multinomial distribution, to model the variation of the whole bacterial community composition in a habitat [41]. In the model, theta is an overdispersion parameter reflecting the variance of each taxon and covariance between taxa. It ranges from 0 to 1, with the higher value representing higher variation (Figure 6). Lowest variation (theta <0.1) was observed for the nine oral sites and stool samples. Two of the three habitats showing highest variation were vaginal sites. Although these sites had the lowest alpha diversity, due to the dominance of *Lactobacillus *in most samples, the high overdispersion resulted from the subset of samples with low *Lactobacillus *abundance. Skin habitats also showed higher overdispersion presumably due to their exposure to the environment. High variability was also observed for urine and preterm baby stool samples as expected since neither of these habitats shows consistent patterns of organisms between subjects.

The numbers of organisms that we detected in samples were largely influenced by the sequence depth and number of sampled individuals (Figure S3B in Additional file 1). However, community variation was not sensitive to sequencing depth. Community variation measured by theta in DM model at 1,000, 3,000, 6,000, and 9,000 read depths was not significantly different (*P *>0.05) (Figure 6).

### Cosmopolitan and endemic features of human microbiota

In microbial ecology, the abundant species are found in many samples, that is, show high prevalence. Similarly, highly abundant genera in the human microbiota were present in higher percentage of subjects, whereas low abundance genera were restricted in distribution. This phenomenon is shown for the anterior nares (Figure 4) and the other body sites (Figure S5A, 5B, 5C in Additional file 1). *Propionibacterium *and *Staphylococcus *are present in all anterior nares samples with the abundance of 23% ± 2.6% and %19 ± 3.0% (m ± sd), respectively. The majority of genera (92%) in the anterior nares are <1% abundance. However, we do not have evidence to suggest that they are noise within the sequence dataset as described above. Interestingly, some of the relatively low abundance genera are also widely distributed across subjects. For example, *Streptococcus *and *Anaerococcus *are both present in >80% of the nasal samples, however, their relative abundances are about 2% ± 0.8 and 2% ± 0.3 on average. This observation was also evident in stool and oral sites. *Coprococcus *in stool, *Fusobacterium *in throat and hard palate, and *Haemophilus *in hard palate are all low abundance and high prevalence. As a result, the cosmopolitan aspect of human microbiota is not limited to high abundant taxa.

On the other hand, a small group of subjects can contain relatively highly abundant genera that are unique to those subjects. For example, the genus *Moraxella *was present in 17.5% of the anterior nares samples with relative abundance of 21.4% ± 3.8 (Figure 4). *Prevotella *identified from stool was highly represented in 39% of the samples (10.8% ± 2.3 of relative abundance) (Figure S5 Additional file 1). The presence of high abundance genera in a subset of the cohort indicates the endemic aspect of human microbiota.

**Figure 7:**
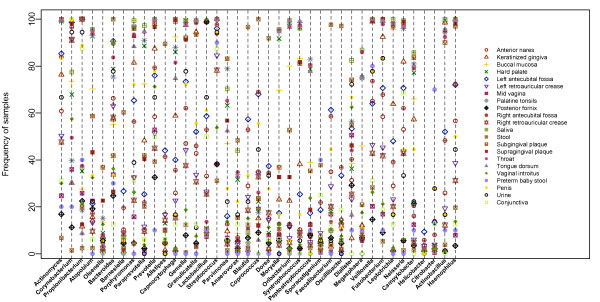
**Taxa distribution across habitats**. Thirty-nine genera present in at least one subject for all 18 HMP habitats are plotted. Each symbol represents a habitat. The y-axis shows the sample prevalence of the genera calculated as the number of samples who harbor the genus divided by the total number of subjects. Using this criterion, 12 of these genera are spread across all 22 habitats.

Analyzing the taxa distribution in a whole body view, we found that there were 39 genera present in all the 18 habitats of the HMP data in at least one subject (Figure 7). Extending this beyond the HMP habitats, and using this same criterion, we found that 12 genera are present in at least one subject of each of the full set of 22 habitats. These 12 genera are found in disparate body sites in samples ranging from the USA to Africa, indicating the extreme cosmopolitan nature of the organisms of these genera. In addition to the intercontinental distribution, these genera were identified in preterm baby, adolescent, and adult samples, suggesting their ubiquity is not limited by age.

There is no single genus present in all habitats of all subjects. *Streptococcus *is the most widespread genus across the whole body with relatively high prevalence. It was found in all the subjects in the oral habitats, nearly all of the skin sites and anterior nares samples, about 50% of vaginal samples, and 30% of stool samples. To investigate whether a locally abundant genus affects its abundance in less dominant sites, the abundance of *Streptococcus *from throat, *Lactobacillus *from posterior fornix, *Bacteroides *from stool were compared with their abundances in the rest of habitats (Figure S6 in Additional file 1). The abundance of *Streptococcus *in throat has moderate correlation with the abundance of *Streptococcus *in other oral habitats except subgingival and supragingval plaques, and it has no correlation with non-oral sites. Similarly, no correlation was found between stool and non-stool site for *Bacteroides*, vaginal and non-vaginal sites for *Lactobacillus*. The strong association of microbes within habitats and lack of association between major habitats is consistent with the findings from another HMP companion paper focusing on the microbial co-occurrence in human bodies [26]. However, both of our conclusions were based on the genus level analysis, while species and strain level analysis may reveal a different picture of the correlation pattern within habitat since the same genus from two different habitats may represent different species.

### Temporal variation of bacterial community

**Figure 8:**
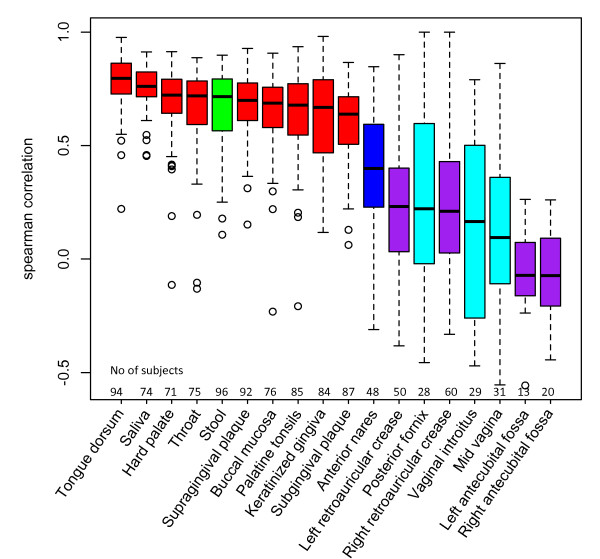
**Community stability over time**. The longitudinal studies were based on 18 habitats from HMP. The similarity of the bacterial communities of each subject between two sampling points was evaluated by Spearman correlation coefficient (y-axis). Oral habitats and stool showed higher correlation between visits; skin and vaginal habitats showed lower correlation between visits. The variation of community stability between subjects is especially high in the skin habitats.

To gain insight into temporal changes of the human microbiome in the 18 HMP body habitats, we evaluated the community similarity between two visits by Spearman correlation (Figure 8). The mean time interval between visits is 212 days (Table S3 in Additional file 2). Bacterial communities in oral and stool habitats have strong correlation (>0.6) between first and second visits, thus representing stable communities, whereas skin/nares and vaginal sites have weak correlation (<0.4) between visits, thus representing variable communities. Furthermore, within a body site, correlation between visits varies greatly from person to person, especially in skin and vaginal sites.

Consistent with previous studies on the dynamics of the vaginal microbiota [42], we also detected a significant change in the relative abundance of *Lactobacillus *between visits in 11 out 29 repeated samples (Figure S7 in Additional file 1). A decrease of *Lactobacillus *was accompanied by an increase in the bacterial vaginosis-associated genera such as *Gardnerella, Prevotella*, and *Atopobium*.

We also observed drastic variation in bacterial communities of the anterior nares between visits. *Moraxella *is a genus that colonizes mucosal surfaces and can give rise to opportunistic infections. It is also one of the notable causes of otitis media and sinusitis. In this dataset, *Moraxella *was detected in 17.5% of the sampled individuals. High variation was detected in nine out of 48 paired samples. For example, in one subject *Moraxella *was 74.3% in abundance in the first visit, but dropped to 0% in the second visit while another subject showed 0% in first visit and 66.2% in second visit (Figure S8 in Additional file 1). The increased abundance of *Moraxella *was accompanied by decreasing abundance in other genera, such as *Staphylococcus, Propionibacterium*, and *Corynebacterium*.

The time interval between two sampling points ranged from 30 days to 359 days and we additionally examined the correlation of the time interval and bacterial community variability. No strong correlation between time interval and the degree of bacterial variability was found (Table S3 in Additional file 2). A weak negative correlation was found in left retroauricular crease and throat.

### Comparison of HMP data with other human microbial data

The HMP seeks to define the baseline of a healthy microbiome. The large datasets produced are potentially of use as healthy controls for other studies. To investigate the utility of HMP data for other studies, we examined the comparability of HMP data with published data. We used the data from a study of the microbiota of lean and obese twins (154 subjects) and a Chinese saliva study of the microbiota in dental caries (45 subjects) (Table S4 in Additional file 2) [43,44]. Reads were reprocessed as had been done for the HMP dataset to exclude bias caused by analysis pipelines. All samples were subsampled to 1,000 reads.

The taxa identified from different projects were relatively consistent. For instance, 72% of the prevalent genera (defined as present in at least 50% of the samples) are present in both HMP saliva samples and Chinese saliva samples. Nine out of 12 phyla identified in HMP stool samples were also identified by the twin study at 1,000 read depth. The three phyla unique to HMP stool samples were present in only 4, 14, and 16 reads in total and thus were quite minor and possibly absent due to sampling rather than biological issues.

Although different studies detected similar prevalent or abundant taxa, the frequency of taxa varied among studies. The frequency of *Bacteroidetes *from HMP data is significantly higher than that in the twin study (Wilcoxon rank sum test, *P *<0.001) (Figure S9 in Additional file 1). Hierarchical clustering of stool samples using the Bray-Curtis dissimilarity was performed as a general comparison. Surprisingly, stool clustering revealed two distinct groups (Figure S10A in Additional file 1) with each cluster based on projects. The HMP saliva samples and the Chinese saliva samples were separated into two project-based clusters with the exception of six caries and two healthy Chinese saliva samples that clustered with HMP healthy samples. Two Chinese caries samples were outliers (Figure S10B in Additional file 1). The clear boundary separating samples by projects suggest significant variation between the bacterial communities in each project. Multiple factors differ between projects (for example, read length, DNA extraction, 16S rRNA region, see Table S4 in Additional file 2), and these in addition to individual variation, demographic difference, and other technical issues [45] can have significant impact on bacterial community structure. The above analyses indicate the challenges for comparisons across projects and suggest that care must be taken in use of HMP data as healthy controls for other projects.

## Discussion

Using the large 16s rRNA gene dataset generated by HMP and other projects, we analyzed the biogeographic patterns of human microbiota from 22 different habitats.

The biodiversity by the 22 habitats representing the human body has implications in human microbial ecology. Each habitat has its own characteristic biodiversity and taxon abundance distribution curve. While there are many contributing factors to each characteristic pattern, this description of the healthy state will contribute to recognition of changes associated with disease. The trend of the biodiversity changes varies with diseases. For example, diversity was reduced in stool habitat with obesity [43], but increased in the vaginal habitat with vaginosis [46]. However, it is not clear whether the disturbed biodiversity is the consequence or the cause of disease. Moreover, compared to classical macro-ecological systems, where healthy ecosystems show high biodiversity, considered important for stability and surviving stress to the system, it is apparent that the situation with the human microbiome is more complex as there is no simple rule relating biodiversity to health. Thus for use of biodiversity as a diagnostic of health or disease, careful analysis of the baseline healthy state in each habitat is required.

Despite the community variation with different habitats, the structures of these communities in human habitats adhere to general ecological rules. For example, most habitats have a few dominant taxa with a large number of individually rare species; the more abundant taxa show the most dispersal among subjects, although some minor taxa are widespread too; habitats showing lower Shannon diversity (vagina, skin) also are among the most variable. It was reported that the vaginal bacterial communities from healthy adults underwent drastic changes over a short period of time [21]. In this study, we also observed the dynamic community structure of the anterior nares. Clinical studies have revealed that the prevalence of pharyngeal colonization and respiratory tract infections caused by *Moraxella catarrhalis *displays seasonal variation and increases in winter [47]. Unfortunately, no detailed seasonal information is available for this dataset. Further study with multiple sampling points at different seasons will provide a better picture of the seasonal effect on the bacterial community in the anterior nares. In general, familiar concepts from macro-ecology appear to apply. Both cosmopolitan and endemic organisms are found in the human microbiome, with many organisms found in multiple habitats, albeit at varying abundance, so the adage 'everything is everywhere, but environment selects' [48] in ecology appears to hold for the human microbiome as well.

Distinguishing rare organisms from noise is challenging. Although methods for denoising data to remove reads with sequencing errors is useful, even after quality filtering and chimera removal there are still artifacts to be addressed such as contamination. Tests based on increasing sequencing depth or prevalence in samples partially address artifacts but still leave aspects unresolved. It should be noted that the presence of *Propinionbacterium *and *Corynebacterium *in stool samples is uncertain for these reasons. More carefully designed controls for multiple sample processing steps are important for more definitive studies on rare organisms.

The goal of defining the normal human bacterial biogeography is to be able to identify specific associations of altered bacterial biogeography with human disease. The high level inter-personal variation of bacterial communities has significant impact for study designs. Host genotype, effects due to viruses, sub-genus, and sub-species level genetic variation, functional equivalence of multiple taxa, and other biological effects all contribute to this variation and will need to be measured and/or controlled in studies seeking disease correlations. In addition, efforts are required to carefully select subjects to preclude bias and impose universal protocols of metagenomic sample preparation and sequencing, as exemplified by the HMP. Metagenomic DNA storage, preparation [45], sequencing, and analysis pipelines vary with different projects, which complicates the comparison between projects, as seen here in comparisons of HMP data with twin study data as well as with Chinese saliva data.

Despite the scale of the dataset in terms of numbers of sequences, the total number of study subjects is still relatively small in epidemiological terms, albeit larger than most previous studies. Efforts to define the appropriate study design to obtain statistical power for conclusions as to effects at different taxonomic levels are underway but are not yet complete. Thus the observations reported here should be taken with this caveat in mind.

Finally, we note that the approach presented here provides an organismal picture of 'who' comprises the human microbiome, potentially useful for recognizing deviations in disease. However, the 16S rRNA gene method involves PCR amplification with degenerate primers that may nevertheless favor some taxa over others. This method also creates chimeric sequences that are largely but not completely removed during data processing. With the decrease of sequencing cost, sequencing large cohorts of samples by WGS is feasible in the future. The taxonomic profiling derived from WGS data (that is, MetaPhlAn) will overcome the above concerns [49]. Furthermore, the taxonomic approach using either 16S rRNA gene or WGS sequences must be complemented by studies of the individual organisms to understand 'what' functional activities they are providing, necessary for a mechanistic understanding of the functioning of the microbiome. The pure focus on the organisms of many studies of the human microbiome leaves this analysis incomplete. As aptly stated by early researchers in the field 'It is what bacteria do rather than what they are that commands attention, since our interest centers in the host rather than the parasite' [50].

## Conclusions

We comprehensively addressed the bacterial biogeography of multiple habitats from a cohort of 279 healthy subjects. What emerges is the beginning of a detailed picture of the character of each of these habitats with respect to aggregate ecological properties such as biodiversity as well as the specifics of their bacterial compositions. Each of these habitats was also analyzed with respect to variation between subjects, variation over time, and correlations between the habitats. While the biogeographic patterns of the human microbiome have been establishing, future investigations should turn the focus from observational study to hypothesis driven study. By testing the current and novel biogeographic theories, we will better understand the underlying mechanisms that govern the observed biogeographic patterns. This in turn will enable us to manipulate our microbiota in clinic for medical benefit.

## Materials and methods

### Ethics statement

Subjects provided written informed consent for screening, enrollment, and specimen collection. The HMP protocol entitled 'HMP-07-001 Human Microbiome Project - Core Microbiome Sample Protocol A' was reviewed and approved by Institutional Review Boards at Washington University in St. Louis, IRB ID#: 201105198 (previously 08-0754) and Baylor College of Medicine, IRB ID#: H-22895. The preterm infant study 'The Neonatal Microbiome and Necrotizing Enterocolitis' was reviewed and approved by the Institutional Review Board at Washington University in St. Louis, HRPO #: 201104267 (previously 09-0652). Parents of infants provided informed consent under HRPO #: 09-0652. The penis samples were obtained as part of the project 'The Young Men's Project' reviewed and approved by the Indiana University Institutional Review Board - 1011004291 (previously 0906-17). The conjunctival samples were collected with permission from the 'Gambian Government/Medical Research Council Unit, The Gambia Joint Ethics Committee' under study number L2011.16. The projects adhered to the regulations of the boards for all experiments and research was conducted according to the principles expressed in the Declaration of Helsinki. Data were analyzed without personal identifiers.

### Sample collection

HMP samples were collected by teams at the Baylor College of Medicine and Washington University in St. Louis based on a sampling strategy developed by HMP. Core Microbiome Sampling Protocol A (HMP-A) was provided by the NIH Roadmap for Medical Research. In brief, 236 healthy adults were included in the HMP analysis. Fifteen habitats comprised of anterior nares, skin (left and right retroauricular crease, left and right antecubital fossa), oral (hard palate, keratinized gingiva, buccal mucosa, subgingval plaque, supragingval plaque, saliva, tongue dorsum, palatine tonsil, and throat), and stool were sampled from both male and female subjects. Female subjects were sampled at three extra sites, vaginal introitus, posterior fornix, and mid vagina. Subjects with vaginal pH >4.5 were excluded from the study. For longitudinal studies, sets of samples from each habitat were collected at two time points (Table S3 in Additional file 2). The mean time interval between the two time points was 212 days. We also included some datasets from other projects. The control group from a trachoma project with collaborators from The Gambia showed no evidence of acute or chronic trachoma and had normal healthy conjunctiva. Those samples provide a view of microbiota in the conjunctiva. Preterm baby stool samples from a neonatal microbiome project [51] contribute to the more complete picture of stool microbiome. Microbiota vary greatly in the early weeks of life, thus a single sampling point was chosen at the age of 4 weeks. No antibiotics were taken 3 weeks before sampling of the preterm baby stool. The urine habitat from healthy controls from the Urethral Microbiome of Adolescent Males project were added to represent other body habitats that were not represented in the HMP samples [7]. The penis microbiome data was also from the Urethral Microbiome of Adolescent Males project. Urine specimens were tested for *C. trachomatis *and *N. gonorrhoeae *infection.

### 16S rRNA gene sequencing process

Sequencing data were produced by the Baylor College of Medicine Human Genome Sequencing Center, the Broad Institute, the Genome Center at Washington University, and the J. Craig Ventor Institute. The quality filtering and trimming, chimera checking were performed as described [24]. In brief, the protocol allows one mismatch in the primer and zero mismatches in the barcode. Chimeric reads were removed using Chimera slayer software [52]. At the beginning of the HMP, investigation on the Mock Community that is composed of known organisms demonstrated that the increased richness was mainly from the chimeric and low quality reads [31]. All the high quality 16S rRNA gene reads were classified from phylum to genus level at a confidence threshold of 0.5 using the Ribosomal Database Project (RDP) Naiive Bayesian Classifier version 2.2, training set 6 [32]. The reads whose taxonomic assignments were <0.5 confidence threshold were assigned to the unclassified group. Samples with <1,000 reads were removed to ensure adequate representation of the community structure. The reads used in this analysis can be downloaded from the Data Analysis and Coordination Center (DACC) website [55] with SRA study ID SRP002395. The metadata of HMP study was submitted by the EMMES Corporation, which serves as the clinical data collection site for the HMP. It can be obtained from dbGaP website [56].

### Reads subsampling

Read subsampling is done using the rarefy function from the Vegan package in R [53]. The random sampling is done without replacement so that the variance of rarefied communities is related to the rarefaction proportion rather than to the size of the sample. To avoid the bias that is potentially caused by different sequencing depths, all the samples were rarefied to 1,000 reads in Figures 3, 5, 7, and 8 and additional files involved comparison across sites. The minimal number of reads among all the samples were used for the analysis of the taxa abundance association between habitats. The taxa relative abundances were calculated as the reads assigned to the taxa divided by the total number of reads (after subsampling) of the sample.

### Accumulation curve

Accumulation curves were used to evaluate the total number of genera in a body habitat and performed using the Vegan Package in R [53]. As mentioned in the read subsampling method, to compare the richness of 22 habitats, 1,000 reads (minimal reads for the sample) were subsampled from each sample for the accumulation curve analysis (Figure S3A in Additional file 1). To assess the overall richness of the 22 habitats, the maximum number of reads for each sample was used (Figure 1). For both accumulation curves, the number of genera was summed stepwise with the accumulation of subjects. Only new genera were added in each step. This process was repeated 500 times randomizing the choice of samples. The average value for each sample point was plotted.

### Bacterial distribution pattern viewed by rank abundance curve

Rank abundance curve analysis was conducted by aggregation of the reads from all samples per habitat, and further normalized to relative abundance. The genera were ranked from highest abundance to the lowest abundance along the x-axis and the corresponding relative abundances were plotted on the y-axis. Analysis was conducted by the Biodiversity package in R [54].

### Measuring community variation by the overdispersion parameter of DM model

To measure single taxon distribution in a given habitat, we computed the range and percentiles of its frequency across all the samples. The overall subject variation was represented by the average variation of all the taxa in a body site and evaluated by the overdispersion parameter  θ in the Dirichlet-multinomial (DM) model. Human metagenomic data with high inter-subject variation follow Dirichlet-multinomial (DM) distribution as shown by us and other independent study recently [40,41]. It is defined as:

$$\text{P}\left(X_{i}=x_{i}\right)=\frac{N_{i}!}{{\text{x}}_{\text{i}1}!\ldots {\text{x}}_{\text{iK}}!}\frac{\prod_{\text{j}=1}^{\text{K}}\prod_{\text{r}=1}^{{\text{x}}_{\text{ij}}}\left\{\pi_{\text{j}}\left(1-\theta \right)+\left(\text{r}-1\right)\theta \right\}}{\prod_{\text{r}=1}^{N_{i}}\left(1-\theta \right)+\left(\text{r}-1\right)\theta },$$

where $x_{ik}$, is the number of reads in subject  i for taxon k, for i=1,…,P with P the total number of subjects and k=1,…,K with K the total number of taxa, $N_{i}=\sum_{\text{j}=1}^{\text{K}}{\text{x}}_{\text{ij}}$is the total number of sequence reads, $\pi =\left\{\pi_{j},j=1,\ldots ,K\right\}$ is the vector containing the mean of the taxa-frequencies, and  θ is an overdispersion parameter. The vector  π provides the average distribution of taxa frequencies across subjects, and the parameter  θ quantifies the variation of taxa frequency.  θ ranges from 0 to 1, if θ=0 it reduces to the multinomial distribution, which means that every sample has the same taxa frequency distribution.  θ is a suitable parameter to quantify the variation of subjects as measured by the taxa abundance distributions. Both  π and  θ can be estimated directly from the data using either the methods of moments or the maximum likelihood estimation method. The above analysis was performed using the R package from [41].

### Diversity comparison

To compare the diversity between habitats, we computed Richness and Shannon index for each individual in the habitats. For 22 habitats, a two sample t-test was performed on paired habitats. The *P *value was adjusted by the Bonferroni method.

### Diversity correlation

Diversity correlations between habitats were performed using the subjects who have samples for both habitats. Due to the purpose of comparison of richness diversity between habitats, samples were subsampled to the minimal number of reads of the two habitats to be compared by random sampling [53]. The correlation of richness diversity of paired habitats was assessed by Spearman correlation coefficient. Bray-Curtis dissimilarity matrix was calculated for each habitat using the same abundance matrix generated for evaluation of richness diversity correlation. Mantel correlation was used to compare the paired distance matrices and assess the beta diversity association.

### Evaluation of community similarity between two visits

Samples with both visit 1 and visit 2 data were used to investigate the temporal variation of the bacterial community for all of the 18 habitats. The similarity of the bacterial community between visits was evaluated by Spearman correlation coefficient.

## Abbreviations

HMP: Human Microbiome Project; OUT: Operational Taxonomic Unit; RDP: Ribosomal Database Project; WGS: Whole Genome Shotgun.

## Competing interests

The authors declare that they have no competing interests.

## Authors' contributions

YZ, HG, KAM, PSLA, GMW, and ES did the analysis. YZ, HG, KAM, PSLA, KMW, TV, MP, WDS, ES, and GMW conceived and designed the experiments. BW, PT, DEN, DF, MJH, and SEB contributed to non-HMP sequence data and coordinated the experimental design. YZ and GMW wrote the paper. All authors read and approved the final manuscript.

## Supplementary Material

Additional file 1 **Figure S1. Distribution of unclassified genera of 22 habitats**. Sequences that could not be classified at RDP confidence threshold 0.5 were assigned to unclassified genera. Unclassified reads account for relatively small proportion of the total reads in the majority of the samples. **Figure S2**. **Phylum profiling of 22 human habitats**. The average relative abundance of phyla in each habitat was measured by the fraction of total 16S rRNA gene sequences. Each color represents a phylum. (**A**) Firmicutes, Actinobacteria, and Proteobacteria are the major phyla identified in human body. (**B**) Phyla accounting for <0.5% of the total phyla are shown. Preterm baby stool in this dataset does not contain low abundance phyla with the 0.5% standard, thus there are no data plotted. The total fractions of the phyla <0.5% in this figure are listed on top of the plot. **Figure S3**. **Accumulation curves at the genus level**. The only difference between Figure S3A and Figure 1 is that all the samples were rarified to 1,000 reads in Figure S3A. The accumulation curves exhibit similar patterns in both figures. Figure S3B shows stool richness at different sequencing depths. Sixty stool samples with >9,000 reads were rarified to 1,000, 3,000, 6,000, and 9,000 reads. Both deep sequencing and a large number of subjects are required to detect all the possible taxa. **Figure S4**. **The association of sequencing depth and sample frequency**. The x-axis shows the rank abundance of each genus and the y-axis shows the number of subjects who share the genus. Sixty stool samples with >9,000 reads were rarified to 1,000, 3,000, 6,000, and 9,000 reads. The points showing the abundance of each genus at different depths are linked by line segments. With increased sequencing depth, the number of subjects who share the same genus, including the minor genera, is increased. **Figure S5. The relative abundances of taxa in each habitat and dispersal among subjects**. Dispersal of a given genus is indicated by sample prevalence of that genus on x-axis. The average relative abundance (m ± se) of each genus is indicated on y-axis. The most abundant genera in general have the highest prevalence. However low abundance genera can also be ubiquitous and high abundance genera can be distributed in a limited number of subjects. Also see Figure 4. **Figure S6**. **The correlation of abundant taxa between their dominant habitats and less dominant sites**. The abundances of *Bacteroides *from stool, *Streptococcus *from throat, and *Lactobacillus *from posterior fornix were compared with abundances in the rest of the habitats. The taxon abundance lacks correlation between major habitats (oral, skin, vaginal, stool), but it shows moderate correlation within the oral and vaginal sites. **Figure S7**. **Dynamics of *Lactobacillus *in vaginal habitats between visits**. The relative abundances of *Lactobacillus *undergo great changes between two visits. Vaginosis related genera (*Gardnerella, Prevotella, Atopobium*) are over-represented after *Lactobacillus *loses its dominance. These subjects were asymptomatic and met the criteria of HMP study. **Figure S8**. **Dynamics of *Moraxella *in anterior nares between visits**. The relative abundance of *Moraxella *(colored orange) varies from 0% to 70% between visits. **Figure S9**. **The relative abundances of *Bacteroidetes *and *Firmicutes *in HMP stool samples and twin study stool samples**. The relative abundances of two major phyla *Bacteroidetes *and *Firmicutes *are plotted. *Bacteroidetes *in HMP stool samples are significantly higher than those in obese and lean group of the twin studies (*P *<0.001). **Figure S10. Cluster analysis of the HMP dataset with data from other studies**. (**A**) Clustering analysis of HMP stool and twin study stool samples. Hierarchical clustering was performed using Bray-Curtis dissimilarity and complete linkage. Red labels represent the HMP samples and blue labels represent twin study samples. (**B**) Clustering analysis of HMP saliva and Chinese saliva samples. Red: HMP sample; green: healthy controls of Chinese saliva samples; blue: Chinese saliva samples from subjects with dental caries. The majority of the samples are clustered by project rather than health status. Click here for file

Additional file 2 **Table S1. *P *values of the pair-wised student's t-test of richness (sheet 1) and Shannon diversity (sheet 2) for 22 habitats. Table S2: The variation of the top 20 most abundant phyla (sheet 1) and genera (sheet 2)**. The range and quartiles of each taxon's relative abundance within a habitat are listed. **Table S3**. **Correlation of time interval and bacterial community variation between visits**. The time intervals between visits for 18 body sites are listed. Temporal stability of bacterial community varies with habitats, indicated by the Spearman correlation coefficient in column 5. There is no strong correlation between the time interval and bacterial variation between visits as shown by the Spearman correlation coefficient in column 7. There is a weak correlation for left retroauricular crease and tongue. **Table S4**. **Comparison of the HMP data with other datasets**. Click here for file
