# Supplementary material for: Biogeography of the ecosystems of the healthy human body
Source: Genome Biol. 2013 Jan 14;14(1):R1. doi: 10.1186/gb-2013-14-1-r1 (PMC4054670; doi:10.1186/gb-2013-14-1-r1)

Figure S1

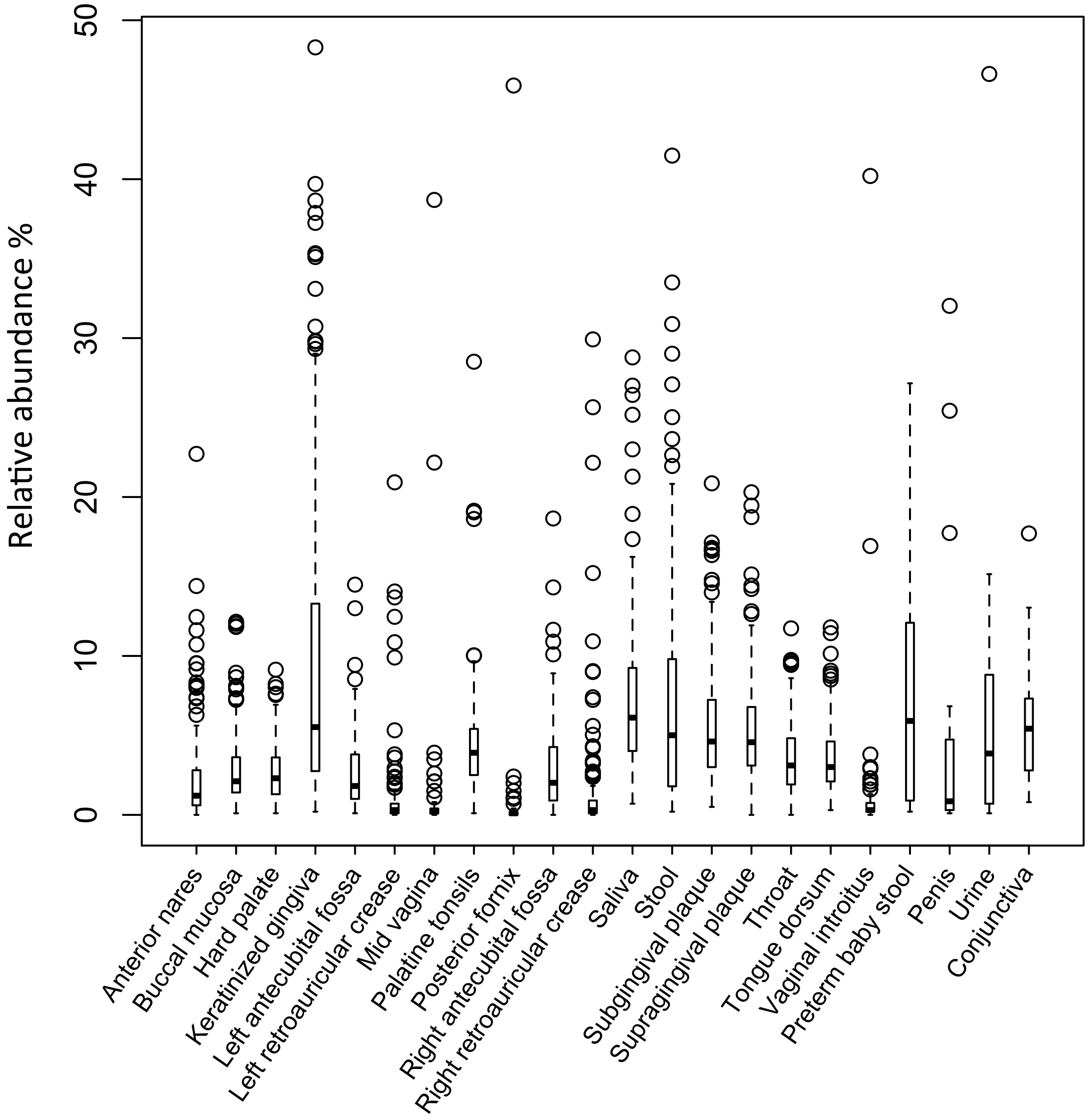
Relative abundance %
0
10
20
30
40
50
Anterior nares
Buccal mucosa
Hard palate
Keratinized gingiva
Left antecubital fossa
Left retroauricular crease
Mid vagina
Palatine tonsils
Posterior fornix
Right antecubital fossa
Right retroauricular crease
Saliva
Stool
Subgingival plaque
Supragingival plaque
Throat
Tongue dorsum
Vaginal introitus
Preterm baby stool
Penis
Urine
Conjunctiva

Figure S2A

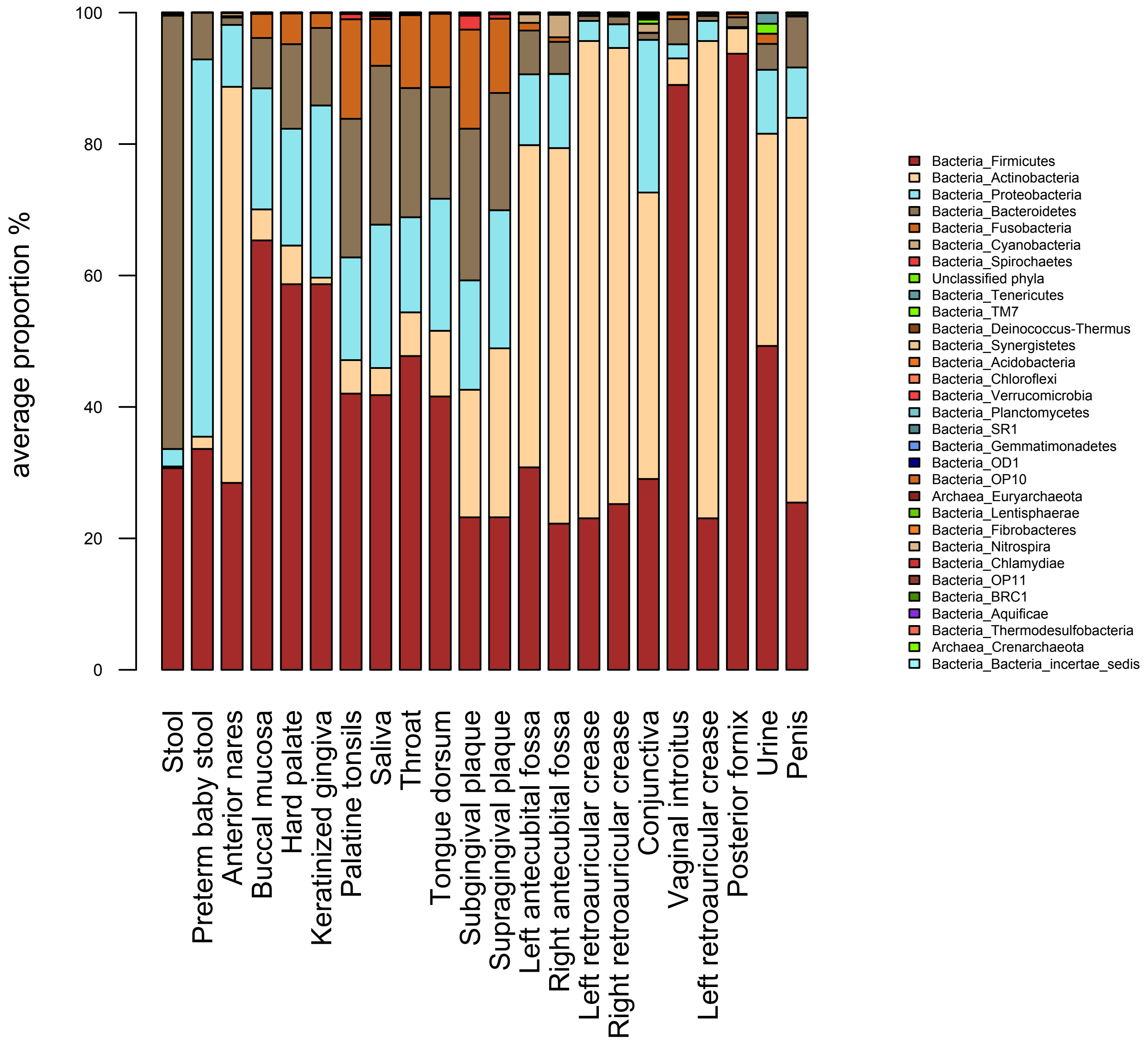

average proportion %
100
80
60
40
20
0
Stool
Preterm baby stool
Anterior nares
Buccal mucosa
Hard palate
Keratinized gingiva
Palatine tonsils
Saliva
Throat
Tongue dorsum
Subgingival plaque
Supragingival plaque
Left antecubital fossa
Right antecubital fossa
Left retroauricular crease
Right retroauricular crease
Conjunctiva
Vaginal introitus
Left retroauricular crease
Posterior fornix
Urine
Penis
Bacteria_Firmicutes
Bacteria_Actinobacteria
Bacteria_Proteobacteria
Bacteria_Bacteroidetes
Bacteria_Fusobacteria
Bacteria_Cyanobacteria
Bacteria_Spirochaetes
Unclassified phyla
Bacteria_Tenericutes
Bacteria_TM7
Bacteria_Deinococcus-Thermus
Bacteria_Synergistetes
Bacteria_Acidobacteria
Bacteria_Chloroflexi
Bacteria_Verrucomicrobia
Bacteria_Planctomycetes
Bacteria_SR1
Bacteria_Gemmatimonadetes
Bacteria_OD1
Bacteria_OP10
Archaea_Euryarchaeota
Bacteria_Lentisphaerae
Bacteria_Fibrobacteres
Bacteria_Nitrospira
Bacteria_Chlamydiae
Bacteria_OP11
Bacteria_BRC1
Bacteria_Aquificae
Bacteria_Thermodesulfobacteria
Archaea_Crenarchaeota
Bacteria_Bacteria_incertae_sedis

Figure S2B

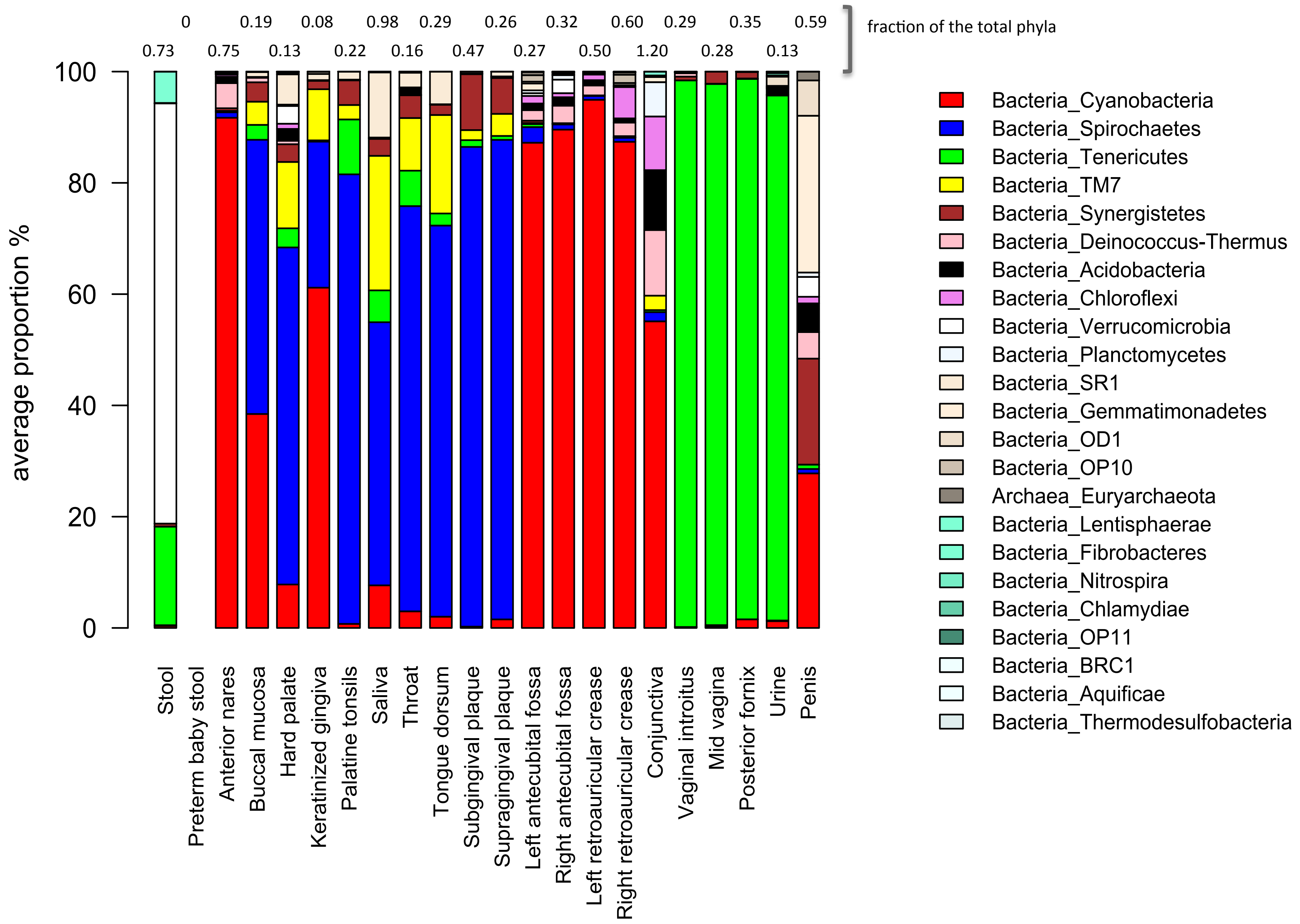

0.73
0
0.75
0.19
0.13
0.08
0.22
0.98
0.16
0.29
0.47
0.26
0.27
0.32
0.50
0.60
1.20
0.29
0.28
0.35
0.13
0.59
fraction of the total phyla
average proportion %
100
80
60
40
20
0
Stool
Preterm baby stool
Anterior nares
Buccal mucosa
Hard palate
Keratinized gingiva
Palatine tonsils
Saliva
Throat
Tongue dorsum
Subgingival plaque
Supragingival plaque
Left antecubital fossa
Right antecubital fossa
Left retroauricular crease
Right retroauricular crease
Conjunctiva
Vaginal introitus
Mid vagina
Posterior fornix
Urine
Penis
Bacteria_Cyanobacteria
Bacteria_Spirochaetes
Bacteria_Tenericutes
Bacteria_TM7
Bacteria_Synergistetes
Bacteria_Deinococcus-Thermus
Bacteria_Acidobacteria
Bacteria_Chloroflexi
Bacteria_Verrucomicrobia
Bacteria_Planctomycetes
Bacteria_SR1
Bacteria_Gemmatimonadetes
Bacteria_OD1
Bacteria_OP10
Archaea_Euryarchaeota
Bacteria_Lentisphaerae
Bacteria_Fibrobacteres
Bacteria_Nitrospira
Bacteria_Chlamydiae
Bacteria_OP11
Bacteria_BRC1
Bacteria_Aquificae
Bacteria_Thermodesulfobacteria

Figure S3A

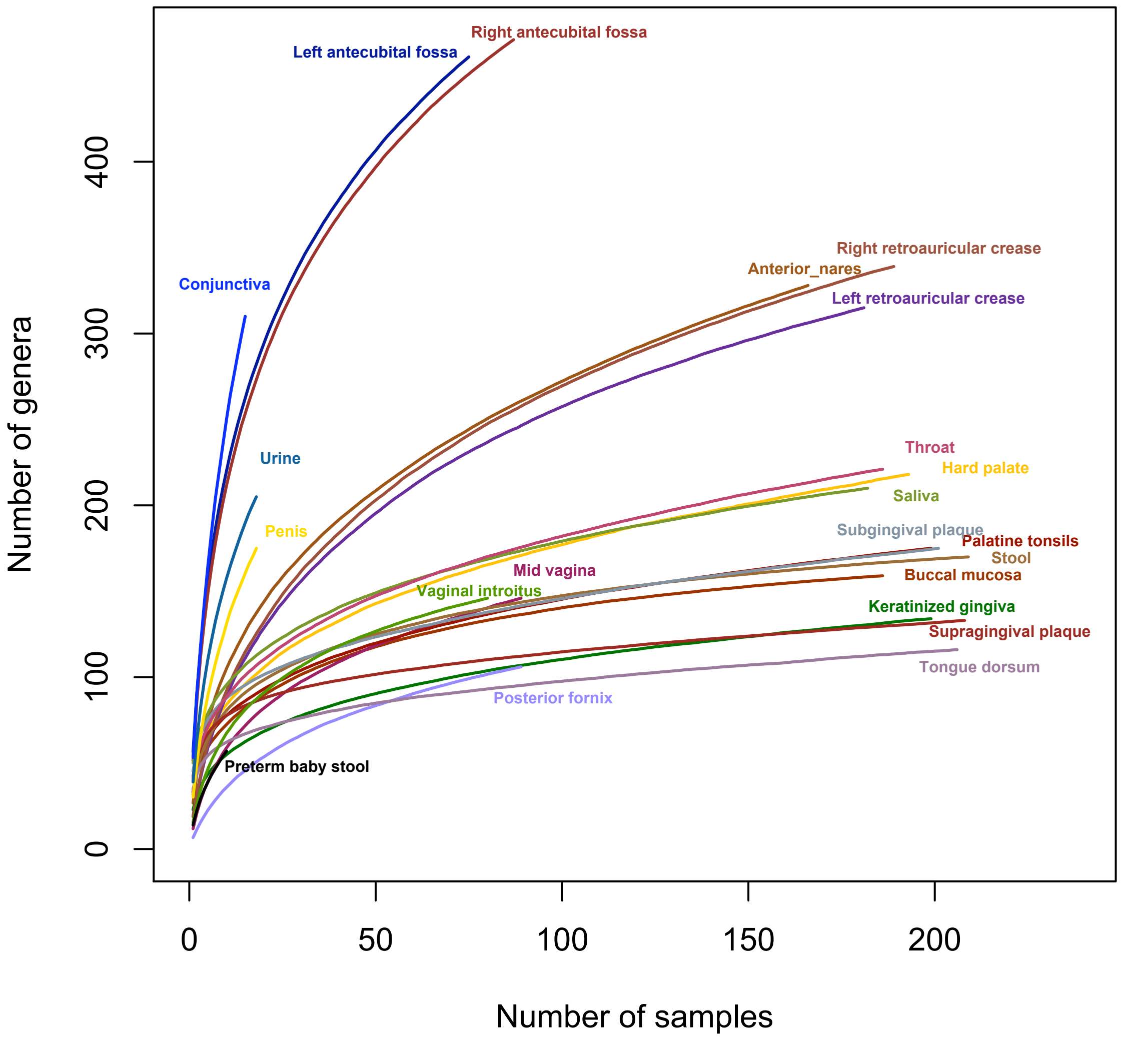

Right antecubital fossa
Left antecubital fossa
Conjunctiva
Right retroauricular crease
Anterior_nares
Left retroauricular crease
Urine
Penis
Throat
Hard palate
Saliva
Subgingival plaque
Palatine tonsils
Stool
Buccal mucosa
Mid vagina
Vaginal introitus
Keratinized gingiva
Supragingival plaque
Tongue dorsum
Posterior fornix
Preterm baby stool
Number of genera
0
100
200
300
400
0
50
100
150
200
Number of samples

Figure S3B

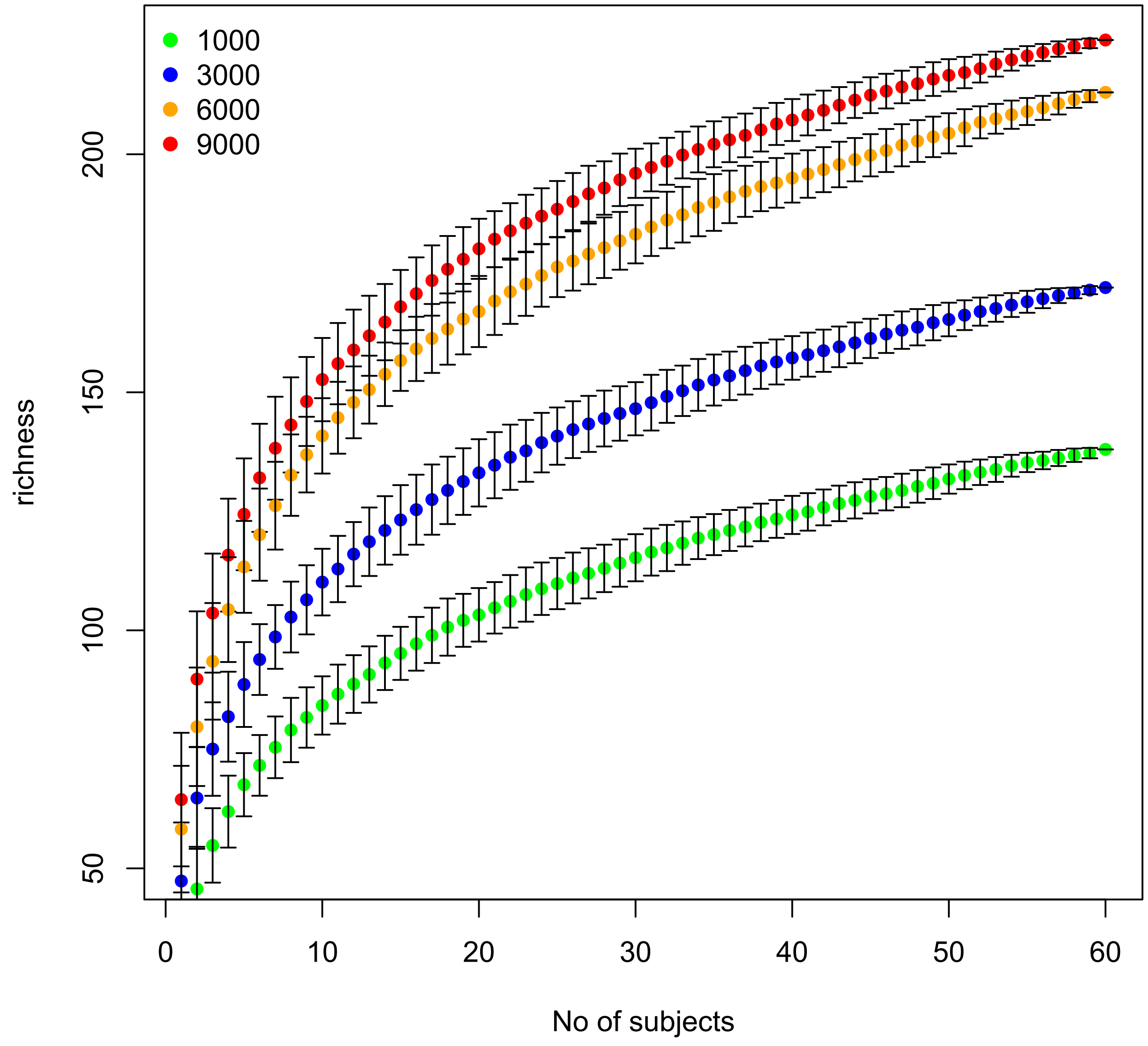
1000
3000
6000
9000
richness
50
100
150
200
0
10
20
30
40
50
60
No of subjects

Figure S4

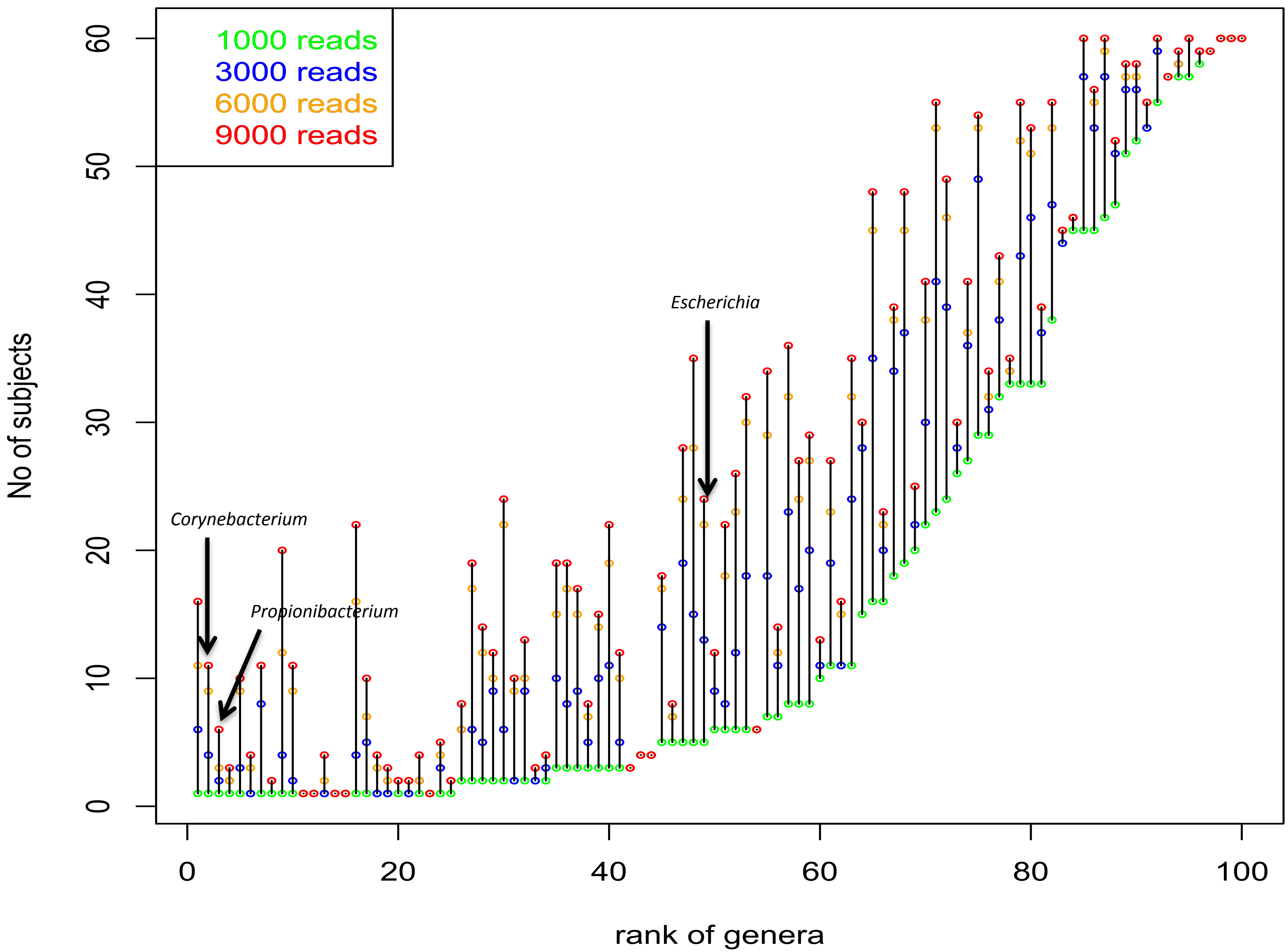
1000 reads
3000 reads
6000 reads
9000 reads
Corynebacterium
Propionibacterium
Escherichia
No of subjects
rank of genera
0
10
20
30
40
50
60
0
20
40
60
80
100

Figure S5A

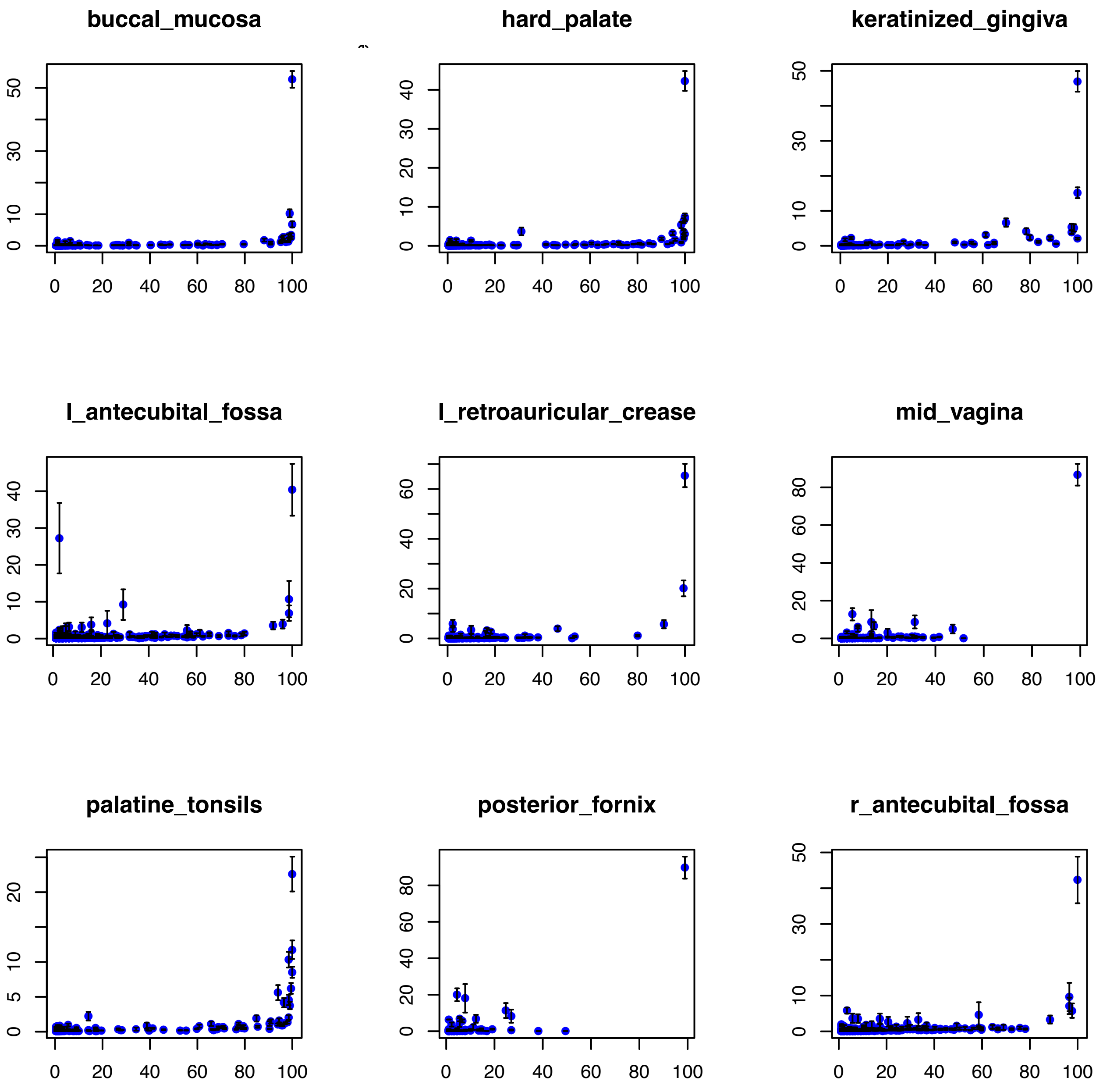
buccal_mucosa
hard_palate
keratinized_gingiva
l_antecubital_fossa
l_retroauricular_crease
mid_vagina
palatine_tonsils
posterior_fornix
r_antecubital_fossa
0 20 40 60 80 100

Figure S5B

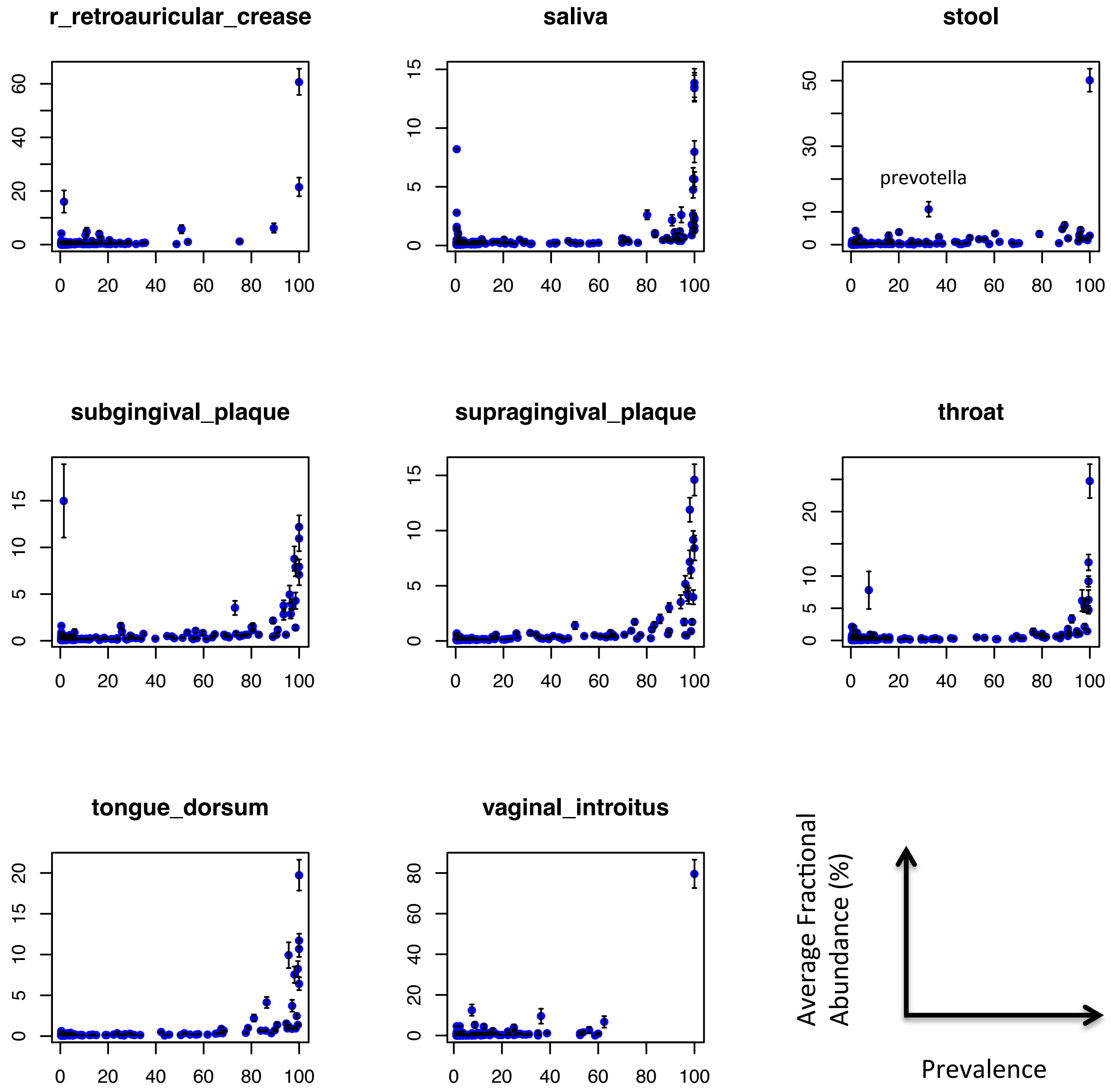
r_retroauricular_crease
saliva
stool
prevotella
subgingival_plaque
supragingival_plaque
throat
tongue_dorsum
vaginal_introitus
Average Fractional Abundance (%)
Prevalence

Figure S5C

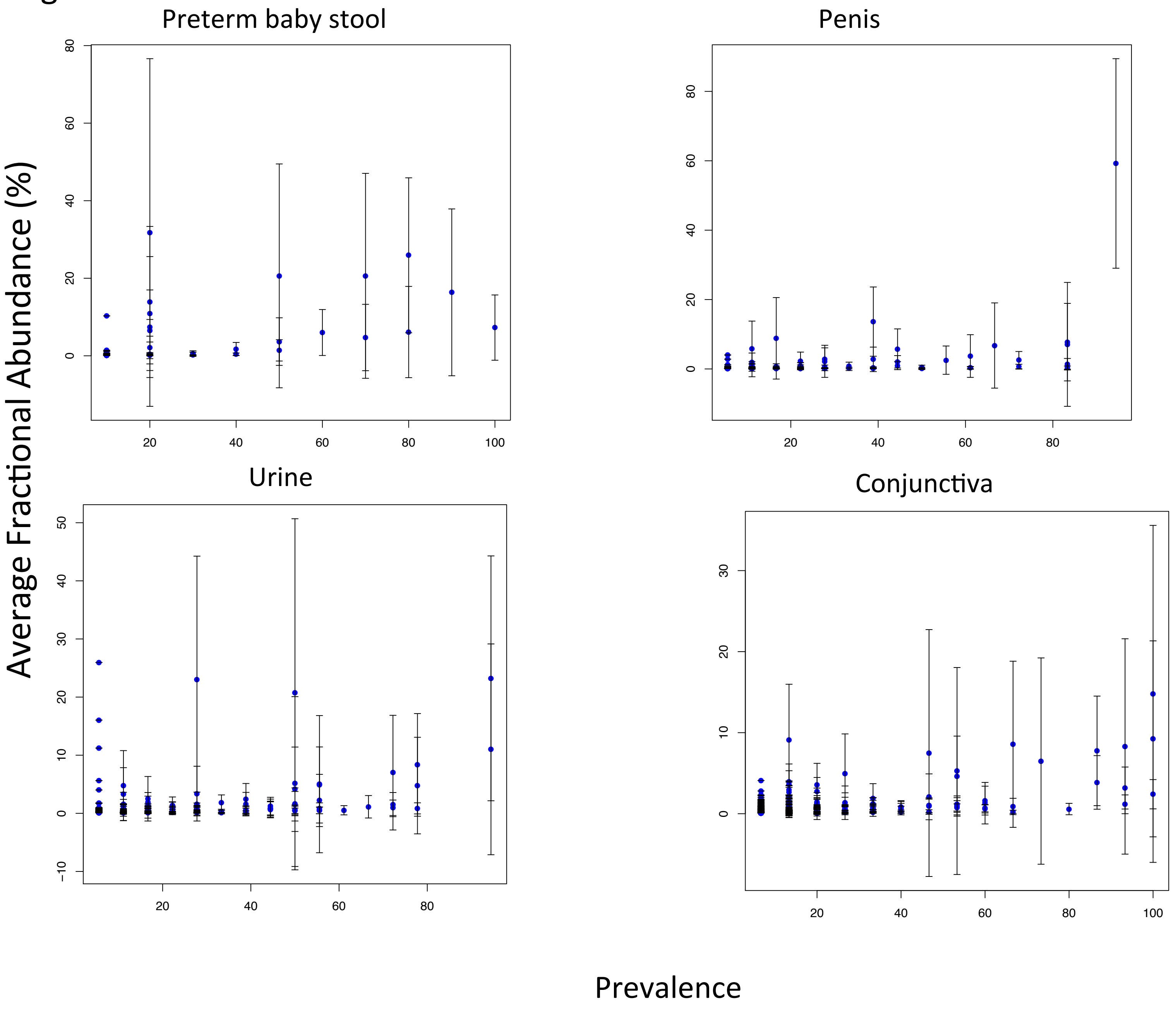

Preterm baby stool
Penis
Urine
Conjunctiva
Average Fractional Abundance (%)
Prevalence

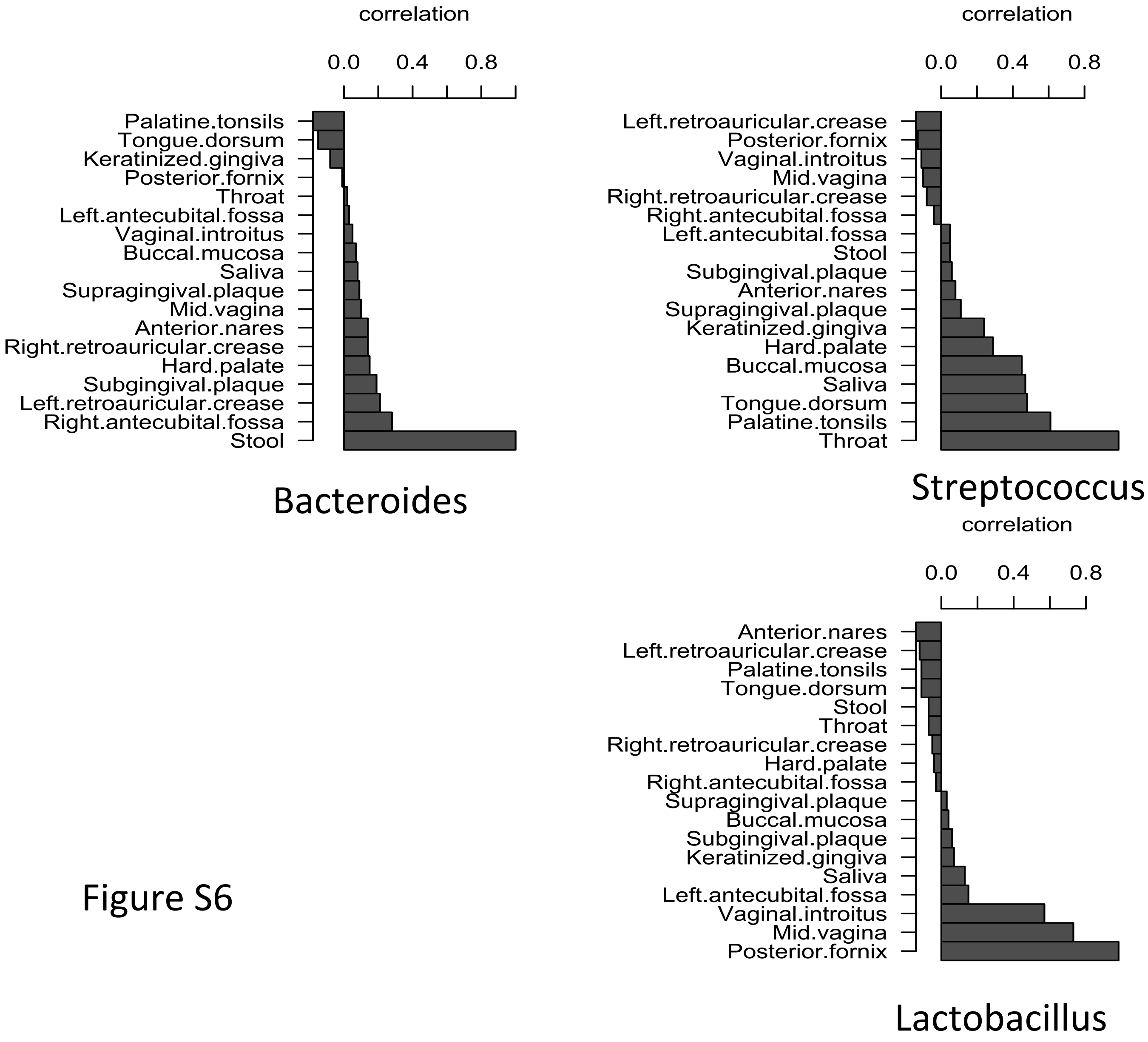
correlation
0.0
0.4
0.8
Palatine.tonsils
Tongue.dorsum
Keratinized.gingiva
Posterior.fornix
Throat
Left.antecubital.fossa
Vaginal.introitus
Buccal.mucosa
Saliva
Supragingival.plaque
Mid.vagina
Anterior.nares
Right.retroauricular.crease
Hard.palate
Subgingival.plaque
Left.retroauricular.crease
Right.antecubital.fossa
Stool
Bacteroides
correlation
0.0
0.4
0.8
Left.retroauricular.crease
Posterior.fornix
Vaginal.introitus
Mid.vagina
Right.retroauricular.crease
Right.antecubital.fossa
Left.antecubital.fossa
Stool
Subgingival.plaque
Anterior.nares
Supragingival.plaque
Keratinized.gingiva
Hard.palate
Buccal.mucosa
Saliva
Tongue.dorsum
Palatine.tonsils
Throat
Streptococcus
correlation
0.0
0.4
0.8
Anterior.nares
Left.retroauricular.crease
Palatine.tonsils
Tongue.dorsum
Stool
Throat
Right.retroauricular.crease
Hard.palate
Right.antecubital.fossa
Supragingival.plaque
Buccal.mucosa
Subgingival.plaque
Keratinized.gingiva
Saliva
Left.antecubital.fossa
Vaginal.introitus
Mid.vagina
Posterior.fornix
Lactobacillus

Figure S6

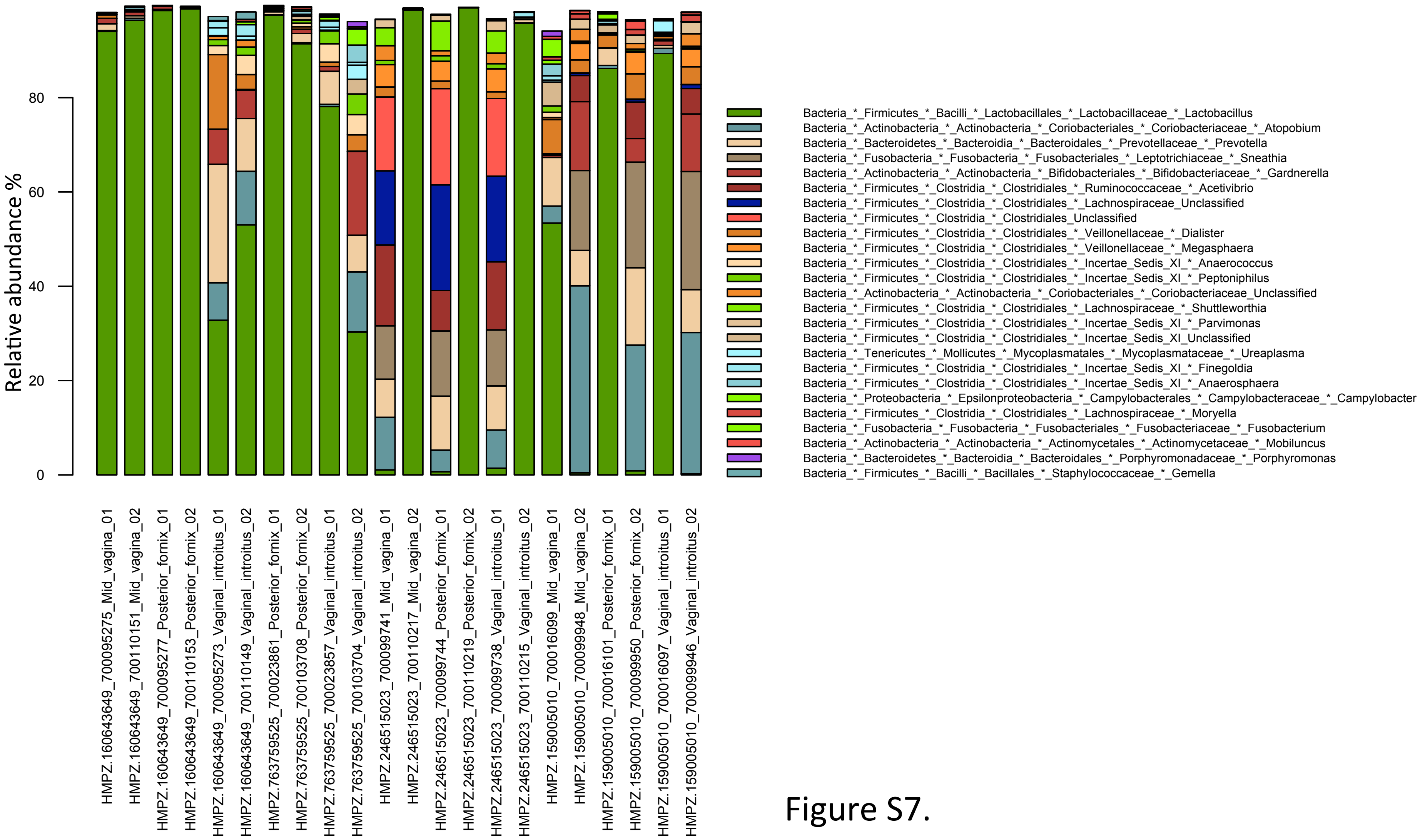
Relative abundance %
0
20
40
60
80
Bacteria_*_Firmicutes_*_Bacilli_*_Lactobacillales_*_Lactobacillaceae_*_Lactobacillus
Bacteria_*_Actinobacteria_*_Actinobacteria_*_Coriobacteriales_*_Coriobacteriaceae_*_Atopobium
Bacteria_*_Bacteroidetes_*_Bacteroidia_*_Bacteroidales_*_Prevotellaceae_*_Prevotella
Bacteria_*_Fusobacteria_*_Fusobacteria_*_Fusobacteriales_*_Leptotrichiaceae_*_Sneathia
Bacteria_*_Actinobacteria_*_Actinobacteria_*_Bifidobacteriales_*_Bifidobacteriaceae_*_Gardnerella
Bacteria_*_Firmicutes_*_Clostridia_*_Clostridiales_*_Ruminococcaceae_*_Acetivibrio
Bacteria_*_Firmicutes_*_Clostridia_*_Clostridiales_*_Lachnospiraceae_Unclassified
Bacteria_*_Firmicutes_*_Clostridia_*_Clostridiales_Unclassified
Bacteria_*_Firmicutes_*_Clostridia_*_Clostridiales_*_Veillonellaceae_*_Dialister
Bacteria_*_Firmicutes_*_Clostridia_*_Clostridiales_*_Veillonellaceae_*_Megasphaera
Bacteria_*_Firmicutes_*_Clostridia_*_Clostridiales_*_Incertae_Sedis_XI_*_Anaerococcus
Bacteria_*_Firmicutes_*_Clostridia_*_Clostridiales_*_Incertae_Sedis_XI_*_Peptoniphilus
Bacteria_*_Actinobacteria_*_Actinobacteria_*_Coriobacteriales_*_Coriobacteriaceae_Unclassified
Bacteria_*_Firmicutes_*_Clostridia_*_Clostridiales_*_Lachnospiraceae_*_Shuttleworthia
Bacteria_*_Firmicutes_*_Clostridia_*_Clostridiales_*_Incertae_Sedis_XI_*_Parvimonas
Bacteria_*_Firmicutes_*_Clostridia_*_Clostridiales_*_Incertae_Sedis_XI_Unclassified
Bacteria_*_Tenericutes_*_Mollicutes_*_Mycoplasmatales_*_Mycoplasmataceae_*_Ureaplasma
Bacteria_*_Firmicutes_*_Clostridia_*_Clostridiales_*_Incertae_Sedis_XI_*_Finegoldia
Bacteria_*_Firmicutes_*_Clostridia_*_Clostridiales_*_Incertae_Sedis_XI_*_Anaerosphaera
Bacteria_*_Proteobacteria_*_Epsilonproteobacteria_*_Campylobacterales_*_Campylobacteraceae_*_Campylobacter
Bacteria_*_Firmicutes_*_Clostridia_*_Clostridiales_*_Lachnospiraceae_*_Moryella
Bacteria_*_Fusobacteria_*_Fusobacteria_*_Fusobacteriales_*_Fusobacteriaceae_*_Fusobacterium
Bacteria_*_Actinobacteria_*_Actinobacteria_*_Actinomycetales_*_Actinomycetaceae_*_Mobiluncus
Bacteria_*_Bacteroidetes_*_Bacteroidia_*_Bacteroidales_*_Porphyromonadaceae_*_Porphyromonas
Bacteria_*_Firmicutes_*_Bacilli_*_Bacillales_*_Staphylococcaceae_*_Gemella
HMPZ.160643649_700095275_Mid_vagina_01
HMPZ.160643649_700110151_Mid_vagina_02
HMPZ.160643649_700095277_Posterior_fornix_01
HMPZ.160643649_700110153_Posterior_fornix_02
HMPZ.160643649_700095273_Vaginal_introitus_01
HMPZ.160643649_700110149_Vaginal_introitus_02
HMPZ.763759525_700023861_Posterior_fornix_01
HMPZ.763759525_700103708_Posterior_fornix_02
HMPZ.763759525_700023857_Vaginal_introitus_01
HMPZ.763759525_700103704_Vaginal_introitus_02
HMPZ.246515023_700099741_Mid_vagina_01
HMPZ.246515023_700110217_Mid_vagina_02
HMPZ.246515023_700099744_Posterior_fornix_01
HMPZ.246515023_700110219_Posterior_fornix_02
HMPZ.246515023_700099738_Vaginal_introitus_01
HMPZ.246515023_700110215_Vaginal_introitus_02
HMPZ.159005010_700016099_Mid_vagina_01
HMPZ.159005010_700099948_Mid_vagina_02
HMPZ.159005010_700016101_Posterior_fornix_01
HMPZ.159005010_700099950_Posterior_fornix_02
HMPZ.159005010_700016097_Vaginal_introitus_01
HMPZ.159005010_700099946_Vaginal_introitus_02

Figure S7.

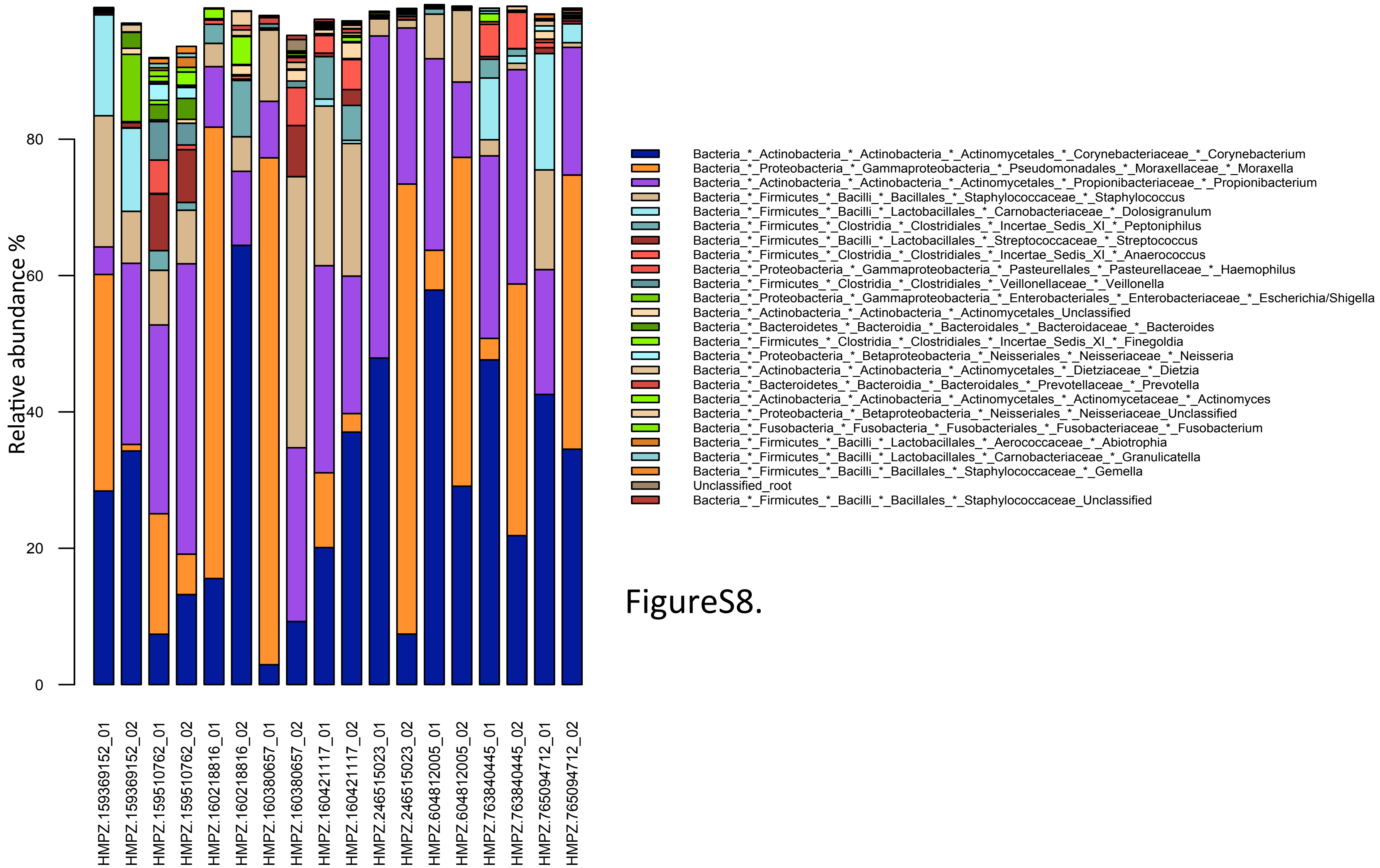
Relative abundance %
0
20
40
60
80
HMPZ.159369152_01
HMPZ.159369152_02
HMPZ.159510762_01
HMPZ.159510762_02
HMPZ.160218816_01
HMPZ.160218816_02
HMPZ.160380657_01
HMPZ.160380657_02
HMPZ.160421117_01
HMPZ.160421117_02
HMPZ.246515023_01
HMPZ.246515023_02
HMPZ.604812005_01
HMPZ.604812005_02
HMPZ.763840445_01
HMPZ.763840445_02
HMPZ.765094712_01
HMPZ.765094712_02
Bacteria_*_Actinobacteria_*_Actinobacteria_*_Actinomycetales_*_Corynebacteriaceae_*_Corynebacterium
Bacteria_*_Proteobacteria_*_Gammaproteobacteria_*_Pseudomonadales_*_Moraxellaceae_*_Moraxella
Bacteria_*_Actinobacteria_*_Actinobacteria_*_Actinomycetales_*_Propionibacteriaceae_*_Propionibacterium
Bacteria_*_Firmicutes_*_Bacilli_*_Bacillales_*_Staphylococcaceae_*_Staphylococcus
Bacteria_*_Firmicutes_*_Bacilli_*_Lactobacillales_*_Carnobacteriaceae_*_Dolosigranulum
Bacteria_*_Firmicutes_*_Clostridia_*_Clostridiales_*_Incertae_Sedis_XI_*_Peptoniphilus
Bacteria_*_Firmicutes_*_Bacilli_*_Lactobacillales_*_Streptococcaceae_*_Streptococcus
Bacteria_*_Firmicutes_*_Clostridia_*_Clostridiales_*_Incertae_Sedis_XI_*_Anaerococcus
Bacteria_*_Proteobacteria_*_Gammaproteobacteria_*_Pasteurellales_*_Pasteurellaceae_*_Haemophilus
Bacteria_*_Firmicutes_*_Clostridia_*_Clostridiales_*_Veillonellaceae_*_Veillonella
Bacteria_*_Proteobacteria_*_Gammaproteobacteria_*_Enterobacteriales_*_Enterobacteriaceae_*_Escherichia/Shigella
Bacteria_*_Actinobacteria_*_Actinobacteria_*_Actinomycetales_Unclassified
Bacteria_*_Bacteroidetes_*_Bacteroidia_*_Bacteroidales_*_Bacteroidaceae_*_Bacteroides
Bacteria_*_Firmicutes_*_Clostridia_*_Clostridiales_*_Incertae_Sedis_XI_*_Finegoldia
Bacteria_*_Proteobacteria_*_Betaproteobacteria_*_Neisseriales_*_Neisseriaceae_*_Neisseria
Bacteria_*_Actinobacteria_*_Actinobacteria_*_Actinomycetales_*_Dietziaceae_*_Dietzia
Bacteria_*_Bacteroidetes_*_Bacteroidia_*_Bacteroidales_*_Prevotellaceae_*_Prevotella
Bacteria_*_Actinobacteria_*_Actinobacteria_*_Actinomycetales_*_Actinomycetaceae_*_Actinomyces
Bacteria_*_Proteobacteria_*_Betaproteobacteria_*_Neisseriales_*_Neisseriaceae_Unclassified
Bacteria_*_Fusobacteria_*_Fusobacteria_*_Fusobacteriales_*_Fusobacteriaceae_*_Fusobacterium
Bacteria_*_Firmicutes_*_Bacilli_*_Lactobacillales_*_Aerococcaceae_*_Abiotrophia
Bacteria_*_Firmicutes_*_Bacilli_*_Lactobacillales_*_Carnobacteriaceae_*_Granulicatella
Bacteria_*_Firmicutes_*_Bacilli_*_Bacillales_*_Staphylococcaceae_*_Gemella
Unclassified_root
Bacteria_*_Firmicutes_*_Bacilli_*_Bacillales_*_Staphylococcaceae_Unclassified

FigureS8.

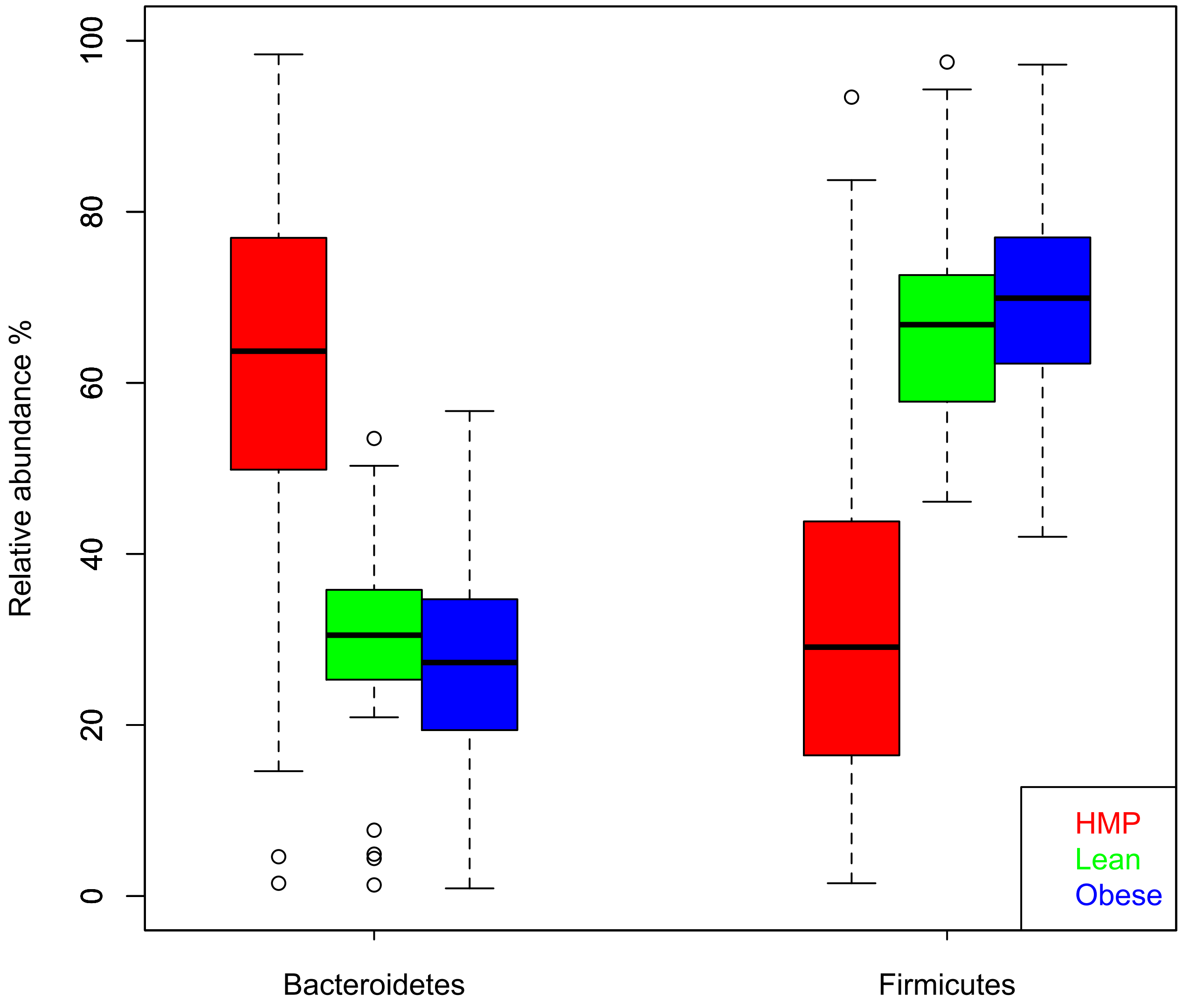
Relative abundance %
0
20
40
60
80
100
Bacteroidetes
Firmicutes
HMP
Lean
Obese

Figure S9

Figure S10

S10A-twin and HMP

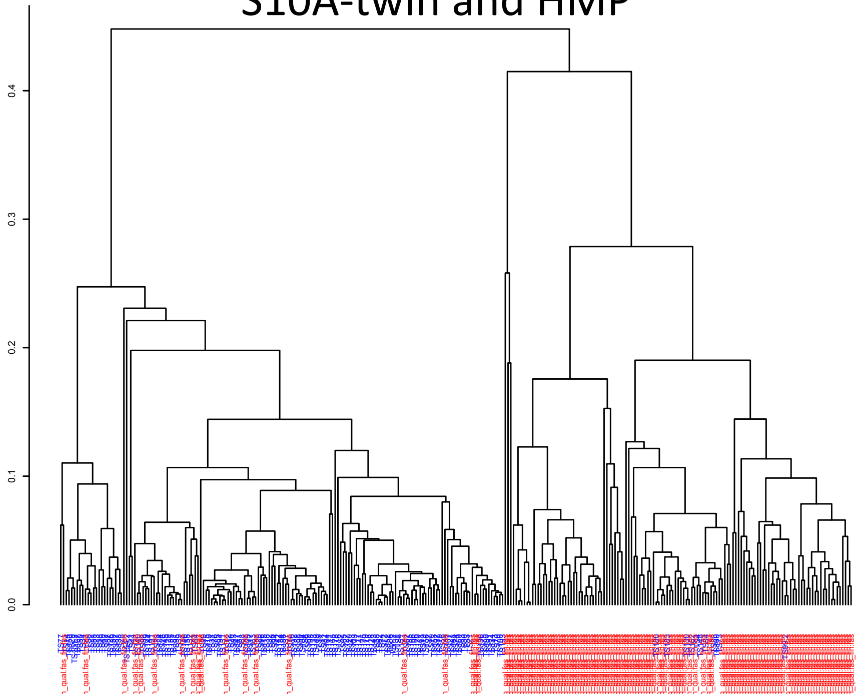

S10B-Chinese caries and HMP

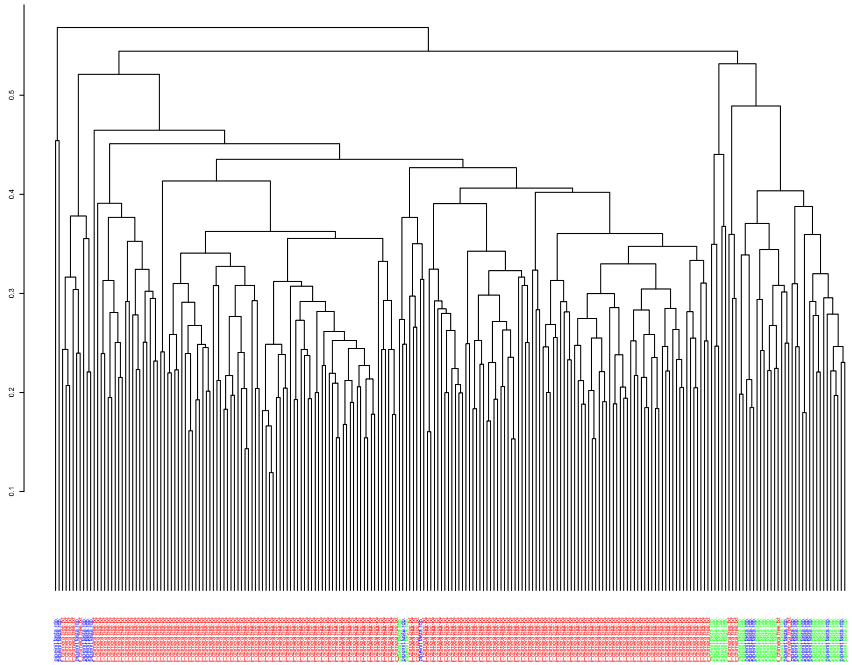

Supplement: Additional file 1 — Figure S1. Distribution of unclassified genera of 22 habitats. Sequences that could not be classified at RDP confidence threshold 0.5 were assigned to unclassified genera. Unclassified reads account for relatively small proportion of the total reads in the majority of the samples. Figure S2. Phylum profiling of 22 human habitats. The average relative abundance of phyla in each habitat was measured by the fraction of total 16S rRNA gene sequences. Each color represents a phylum. (A) Firmicutes, Actinobacteria, and Proteobacteria are the major phyla identified in human body. (B) Phyla accounting for <0.5% of the total phyla are shown. Preterm baby stool in this dataset does not contain low abundance phyla with the 0.5% standard, thus there are no data plotted. The total fractions of the phyla <0.5% in this figure are listed on top of the plot. Figure S3. Accumulation curves at the genus level. The only difference between Figure S3A and Figure 1 is that all the samples were rarified to 1,000 reads in Figure S3A. The accumulation curves exhibit similar patterns in both figures. Figure S3B shows stool richness at different sequencing depths. Sixty stool samples with >9,000 reads were rarified to 1,000, 3,000, 6,000, and 9,000 reads. Both deep sequencing and a large number of subjects are required to detect all the possible taxa. Figure S4. The association of sequencing depth and sample frequency. The x-axis shows the rank abundance of each genus and the y-axis shows the number of subjects who share the genus. Sixty stool samples with >9,000 reads were rarified to 1,000, 3,000, 6,000, and 9,000 reads. The points showing the abundance of each genus at different depths are linked by line segments. With increased sequencing depth, the number of subjects who share the same genus, including the minor genera, is increased. Figure S5. The relative abundances of taxa in each habitat and dispersal among subjects. Dispersal of a given genus is indicated by sample prevalence of that [file gb-2013-14-1-r1-S1.PDF]
